# HrrSA orchestrates a systemic response to heme and determines prioritization of terminal cytochrome oxidase expression

**DOI:** 10.1093/nar/gkaa415

**Published:** 2020-05-26

**Authors:** Marc Keppel, Max Hünnefeld, Andrei Filipchyk, Ulrike Viets, Cedric-Farhad Davoudi, Aileen Krüger, Christina Mack, Eugen Pfeifer, Tino Polen, Meike Baumgart, Michael Bott, Julia Frunzke

**Affiliations:** Institute of Bio- und Geosciences, IBG-1: Biotechnology, Forschungszentrum Jülich, 52425 Jülich, Germany; Institute of Bio- und Geosciences, IBG-1: Biotechnology, Forschungszentrum Jülich, 52425 Jülich, Germany; Institute of Bio- und Geosciences, IBG-1: Biotechnology, Forschungszentrum Jülich, 52425 Jülich, Germany; Institute of Bio- und Geosciences, IBG-1: Biotechnology, Forschungszentrum Jülich, 52425 Jülich, Germany; Institute of Bio- und Geosciences, IBG-1: Biotechnology, Forschungszentrum Jülich, 52425 Jülich, Germany; Institute of Bio- und Geosciences, IBG-1: Biotechnology, Forschungszentrum Jülich, 52425 Jülich, Germany; Institute of Bio- und Geosciences, IBG-1: Biotechnology, Forschungszentrum Jülich, 52425 Jülich, Germany; Microbial Evolutionary Genomics, Institute Pasteur, 75015 Paris, France; Institute of Bio- und Geosciences, IBG-1: Biotechnology, Forschungszentrum Jülich, 52425 Jülich, Germany; Institute of Bio- und Geosciences, IBG-1: Biotechnology, Forschungszentrum Jülich, 52425 Jülich, Germany; Institute of Bio- und Geosciences, IBG-1: Biotechnology, Forschungszentrum Jülich, 52425 Jülich, Germany; Institute of Bio- und Geosciences, IBG-1: Biotechnology, Forschungszentrum Jülich, 52425 Jülich, Germany

## Abstract

Heme is a multifaceted molecule. While serving as a prosthetic group for many important proteins, elevated levels are toxic to cells. The complexity of this stimulus has shaped bacterial network evolution. However, only a small number of targets controlled by heme-responsive regulators have been described to date. Here, we performed chromatin affinity purification and sequencing to provide genome-wide insights into *in vivo* promoter occupancy of HrrA, the response regulator of the heme-regulated two-component system HrrSA of *Corynebacterium glutamicum*. Time-resolved profiling revealed dynamic binding of HrrA to more than 200 different genomic targets encoding proteins associated with heme biosynthesis, the respiratory chain, oxidative stress response and cell envelope remodeling. By repression of the extracytoplasmic function sigma factor *sigC*, which activates the *cydABCD* operon, HrrA prioritizes the expression of genes encoding the cytochrome *bc*_1_-*aa*_3_ supercomplex. This is also reflected by a significantly decreased activity of the cytochrome *aa*_3_ oxidase in the Δ*hrrA* mutant. Furthermore, our data reveal that HrrA also integrates the response to heme-induced oxidative stress by activating *katA* encoding the catalase. These data provide detailed insights in the systemic strategy that bacteria have evolved to respond to the versatile signaling molecule heme.

## INTRODUCTION

Heme (iron bound protoporphyrin IX) is a versatile molecule that is synthesized and used by virtually all aerobic eukaryotic and prokaryotic cells (1). It serves as the prosthetic group of hemoglobins, hydroxylases, catalases, peroxidases and cytochromes (2) and is therefore essential for many cellular processes, such as electron transfer, respiration and oxygen metabolism (3). Furthermore, salvaged heme represents the most important iron source for a variety of pathogenic bacteria (4,5), and also non-pathogenic bacteria can meet their iron demand by degradation of environmental heme. This becomes evident from the diverse set of heme uptake systems and heme oxygenases that catalyze the degradation of the protoporphyrin ring to biliverdin and the concomitant release of carbon monoxide and iron (6).

While heme represents an essential cofactor for a variety of proteins, this molecule also exhibits severe toxicity at high concentrations. Therefore, organisms have evolved sophisticated regulatory networks to tightly control heme uptake, detoxification (including export), synthesis and degradation (4). Several heme-regulated transcription factors have been described, including the heme activator protein (Hap) 1, which is an activator of genes required for aerobic growth of the yeast *Saccharomyces cerevisiae* (7); the transcription factor BACH1 (BTB and CNC homology 1), conserved in mammalian cells (8,9); and the rhizobial Irr protein, which is a heme-regulated member of the Fur family of transcriptional regulators (10–12).

In Gram-positive bacteria, two-component systems (TCSs) appear to play a prevalent role in heme-responsive signaling (13,14), as exemplified by the heme sensor system HssRS of *Staphylococcusaureus* and *Bacillusanthracis*, which controls the expression of the *hrtBA* operon, encoding a heme efflux system in both species (15,16). Remarkably, several members of the *Corynebacteriaceae* family, including the human pathogen *Corynebacterium diphtheriae* and the biotechnological platform strain *Corynebacterium glutamicum*, have two paralogous TCSs, namely, HrrSA and ChrSA, dedicated to heme-responsive control of gene expression (17–20). The kinases HrrS and ChrS were recently shown to perceive transient changes in heme availability by direct intramembrane interactions with heme (21,22). Heme binding triggers autophosphorylation of the sensor kinase, followed by transfer of the phosphoryl group to the cognate response regulators HrrA and ChrA. In *C. glutamicum*, significant cross-phosphorylation was observed between the closely related systems; however, this crosstalk is proofread by a highly specific phosphatase activity of the kinases toward the cognate response regulators under non-inducing conditions (23). While the ChrSA system appears to be mainly involved in rapid activation of the HrtBA detoxification system (19), previous data suggest that HrrSA coordinates a homeostatic response to heme (18). In recent studies, six direct target operons have been described for HrrA, including genes encoding enzymes involved in heme synthesis (*hemE*, *hemA* and *hemH*), heme utilization (*hmuO*, encoding a heme oxygenase) and the *ctaE-qcrCAB* operon, encoding components of the heme-containing cytochrome *bc*_1_-*aa*_3_ supercomplex of the respiratory chain (18). Expression of *hrrA* as well as *hmuO* is, furthermore, repressed by the global iron-dependent regulator DtxR in *C. glutamicum* under conditions of sufficient iron supply (24,25) thereby linking iron and heme regulatory networks in this organism.

The branched electron transport chain of *C. glutamicum* consists of the cytochrome *bc*_1_-*aa*_3_ supercomplex (encoded by *ctaD*, the *ctaCF* operon, and the *ctaE*-*qcrCAB* operon) and the cytochrome *bd* oxidase, encoded by the first two genes of the *cydABDC* operon (26). Although both the cytochrome *aa*_3_ oxidase and the *bd* oxidase are involved in the establishment of a proton-motive force, the *aa*_3_ oxidase is an active proton pump that is responsible for the increased proton translocation number (6 H^+^/2 e^−^) of the cytochrome *bc*_1_-*aa*_3_ supercomplex compared to that of the *bd* oxidase (2 H^+^/2 e^−^) (26). The presence of the cytochrome *bc*_1_-*aa*_3_ supercomplex is a characteristic feature of almost all actinobacteria, because members of this phylum lack a soluble cytochrome *c* and instead harbor a diheme cytochrome *c*_1_ that directly shuttles electrons from the *bc*_1_ complex to the *aa*_3_ oxidase (27–32). Furthermore, both terminal oxidases differ in heme content, as the *bc*_1_-*aa_3_* supercomplex harbors six heme molecules, while the *bd* oxidase harbors only three. Surprisingly, not much is known about the regulation of terminal oxidases in *C. glutamicum*. In addition to the described activation of the *ctaE-qcr* operon by HrrA, the hydrogen peroxide-sensitive regulator OxyR was described as a repressor of the *cydABCD* operon (33,34). Furthermore, the ECF sigma factor SigC (σ^C^) activates expression of the *cydABCD* operon (33,35). For σ^C^, a speculated stimulus is a defective electron transfer in the *aa*_3_ oxidase (35) and such a defect was observed under copper-deprivation or when heme *a* insertion was disturbed, which resulted in activation of the σ^C^ regulon (36,37).

Interestingly, the regulons of prokaryotic heme regulators described thus far comprise only a low number of direct target genes, which are mostly involved in heme export (e.g. *hrtBA*) or degradation (*hmuO*). This current picture of prokaryotic heme signaling, however, does not match the complexity of the cellular processes influenced by heme. In this study, we performed a time-resolved and genome-wide binding profiling of HrrA in *C. glutamicum* using chromatin affinity purification and sequencing (ChAP-Seq) of HrrA in *C. glutamicum* showing the transient HrrA promoter occupancy of more than 200 genomic targets in response to heme. The obtained results emphasize that HrrSA is a global regulator of heme homeostasis, which also integrates the response to oxidative stress and cell envelope remodeling. Transcriptome analysis (RNA-Seq) at different time points after heme induction revealed HrrA to be an important regulator of the respiratory chain by coordinating the expression of components of both quinol oxidation branches as well as menaquinol reduction. Remarkably, HrrA was found to prioritize the expression of operons encoding the cytochrome *bc*_1_-*aa*_3_ supercomplex by repressing *sigC* expression.

## MATERIALS AND METHODS

### Bacterial strains and growth conditions

Bacterial strains used in this study are listed in Supplementary Table S1. The *C. glutamicum* strain ATCC 13032 was used as wild-type (29) and cultivations were performed in liquid BHI (brain heart infusion, Difco BHI, BD, Heidelberg, Germany), as complex medium or CGXII (38) containing 2% (w/v) glucose as minimal medium. The cells were cultivated at 30°C; if appropriate, 25 μg/ml kanamycin was added. *Escherichiacoli* (DH5α and BL21 (DE3)) was cultivated in Lysogeny Broth (Difco LB, BD, Heidelberg, Germany) medium at 37°C in a rotary shaker and for selection, 50 μg/ml kanamycin was added to the medium.

### Recombinant DNA work and cloning techniques

Cloning and other molecular methods were performed according to standard protocols (39). As template, chromosomal DNA of *C. glutamicum* ATCC 13032 was used for polymerase chain reaction (PCR) amplification of DNA fragments and was prepared as described previously (40). All sequencing and synthesis of oligonucleotides was performed by Eurofins Genomics (Ebersberg, Germany). For ChAP sequencing, the native *hrrA* was replaced with a twin-strep-tagged version of this gene using a two-step homologous recombination system. This system is based on the suicide vector pK19 *mob-sacB* (41,42), containing 500 bps flanking each site of the targeted sequence inside the *C. glutamicum* genome. The pK19*mob-sacB hrrA*-C-*twinstrep* plasmid was constructed using Gibson assembly of PCR products (primers indicated in Supplementary Table S2) and the cut pK19 vector (43).

### ChAP-Seq — sample preparation

The preparation of DNA for ChAP sequencing was adapted from (44). The *C. glutamicum* strain ATCC 13032::*hrrA*-C-*twinstrep* was used for the time series experiment. A preculture was inoculated in liquid BHI medium from a fresh BHI agar plate and incubated for 8–10 h at 30°C in a rotary shaker. Subsequently, cells were transferred into a second preculture in CGXII medium containing 2 % (w/v) glucose and 0 μM FeSO_4_ to starve the cells from iron. Protocatechuic acid (PCA), which was added to the medium, allowed the uptake of trace amounts of iron. From an overnight culture, six main cultures were inoculated to an OD_600_ of 3.0 in 1 l CGXII medium containing 4 μM hemin as sole iron source. For the time point t = 0, the cells were added to 1 l fresh CGXII containing no additional iron source. After 0, 0.5, 2, 4, 9 and 24 h, cells corresponding to an OD_600_ of 3.0 in 1 l were harvested by centrifugation at 4°C, 5000 × *g* and washed once in 20 ml CGXII. Subsequently, the cell pellet was resuspended in 20 ml CGXII containing 1 % (v/v) formaldehyde to crosslink the regulator protein to the DNA. After incubation for 20 min at RT, the cross linking was stopped by addition of glycine (125 mM), followed by additional incubation of 5 min at RT. After that, the cells were washed three times in buffer A (100 mM Tris–HCl, 1 mM ethylenediaminetetraacetic acid (EDTA), pH = 8.0) and the pellets stored overnight at −80°C. For cell disruption, the pellet was resuspended in buffer A containing ‘cOmplete’ protease inhibitor cocktail (Roche, Germany) and disrupted using a French press cell (SLM Ainco, Spectronic Instruments, Rochester, NY, USA) five times at 207 MPa. The DNA was fragmented to ∼500 bp by sonication (Branson Sonifier 250, Branson Ultrasonics Corporation, CT, USA) and the supernatant was collected after ultra-centrifugation (150 000 × *g*, 4°C, 1 h). The DNA bound by the twin-Strep-tagged HrrA protein was purified using Strep-Tactin XT Superflow column material (IBA Lifesciences, Göttingen, Germany) according to the supplier's manual (applying the gravity flow protocol, 1.5 ml column volume). Washing of the column was performed with buffer W (100 mM Tris–HCl, 1 mM EDTA, 150 mM NaCl, pH 8,0) and the tagged protein was eluted with buffer E (100 mM Tris–HCl, 1 mM EDTA, 150 mM NaCl, pH 8.0, added 50 mM D-Biotin). After purification, 1 % (w/v) sodium dodecyl sulphate was added to the elution fractions and the samples were incubated overnight at 65°C. For the digestion of protein, 400 μg/ml Proteinase K (AppliChem GmbH, Darmstadt, Germany) was added and incubated for 3 h at 55°C. Subsequently, the DNA was purified as following: Roti-Phenol/Chloroform/Isoamyl alcohol (Carl Roth GmbH, Karlsruhe, Germany) was added to the samples in a 1:1 ratio and the organic phase was separated using Phase Lock Gel (PLG) tubes (VWR International GmbH, Darmstadt, Germany) according to the supplier's manual. Afterwards, the DNA was precipitated by adding ice-cold ethanol (to a conc. of 70 % (v/v) and centrifugation at 16 000 × *g*, 4°C for 10 min. The DNA was washed with ice-cold 70 % (v/v) ethanol, then dried for 3 h at 50°C and eluted in dH_2_O.

### ChAP-Seq — sequencing and peak discovery

The obtained DNA fragments of each sample (up to 2 μg) were used for library preparation and indexing using the TruSeq DNA PCR-free sample preparation kit according to the manufacturer's instruction, yet skipping fragmentation of the DNA and omitting the DNA size selection steps (Illumina, Chesterford, UK). The resulting libraries were quantified using the KAPA library quant kit (Peqlab, Bonn, Germany) and normalized for pooling. Sequencing of pooled libraries was performed on a MiSeq (Illumina) using paired-end sequencing with a read-length of 2 × 150 bases. Data analysis and base calling were accomplished with the Illumina instrument software and stored as fastq output files. The sequencing data obtained for each sample were imported into CLC Genomics Workbench (Version 9, Qiagen Aarhus A/S, Aarhus, Denmark) for trimming and base quality filtering. The output was mapped to accession NC_003450.3 as *C. glutamicum* reference genome (29). Genomic coverage was convoluted with second order Gaussian kernel. The kernel was truncated at 4 sigmas (that is all kernel values positioned further then 4 sigmas from the center were set to zero) and expanded to the ‘expected peak width’. The expected peak width was estimated via the following procedure: (i) all the peaks higher than 3-fold mean coverage were detected. (ii) Points at which their coverage dropped below $\frac{1}{2}$ of the maximal peak height were found and the distance between them was considered as a peak width. (iii) The ‘estimated peak width’ was set equal to the median peak width. The convolution profile was scanned in order to find points where first derivative changes its sign from positive to negative (Supplementary Figure S1). Each such point was considered as a potential peak and was assigned with a convolution score (that is convolution with second order Gaussian kernel centred at the peak position). Furthermore, we explored the distribution of the convolution scores. It appeared to resemble normal distribution, but with a heavy right tail. We assumed that this distribution is indeed bimodal of normal distribution (relatively low scores) representing ‘noise’ and a distribution of ‘signal’ (relatively high scores). We fit the Gaussian curve to the whole distribution (via optimize.fit function from SciPy package; *http://www.scipy.org/*) and set a score thresholds equal mean + 4 sigmas of the fitted distribution. Further filtering with this threshold provided estimated FDR (false discovery rate) of 0.004–0.013 depending on a sample. Filtered peaks were normalized to allow inter-sample comparisons. Sum of coverages of the detected peaks was negated from the total genomic coverage. The resulting difference was used as normalization coefficient; that is peak intensities were divided by this coefficient.

### ChAP-Seq — estimation of confidence intervals

To compare peak intensities between the samples, we assessed the significance levels of the detected intensity values by an extensive *in silico* simulation of ChAP-seq experiments along with further peak-detection analyses.

The simulation consisted of the following steps: The reads were artificially generated from *C. glutamicum* genome (NC_003450.3) with the error rate (number of nucleotide mismatches) equal to the average error rate of the real HrrA ChAP-seq reads (estimated from the mapping statistics). The reads were taken from randomly selected spots in the genome (simulation of the non-peak coverage) and from the regions of the detected HrrA binding peaks with the probabilities proportional to the original peak intensities. Thus, we tried to emulate the original binding architecture. We also added a small amount (10% of the total simulated reads) of the sequences heavily affected by mismatches (25% mismatches for the original *C. glutamicum* sequences), as we wanted to account for around 10% of the unmapped reads in the original HrrA ChAP-seq experiments. Finally, the simulated reads were subjected to the computational peak-detection pipeline with the same parameters as in the original analyses. As a result, we obtained the peak intensity values for the detected peaks.

In total, we simulated 200 ChAP-seq samples, each containing 1.14M reads (the average amount of reads in the original samples). For each of the detected peaks we estimated the variation of the reported peak intensity among all the simulations. That is, for each peak intensity we estimated 0.95 confidence interval, as a difference between 97.5 and 2.5 percentiles. We discovered a strong positive correlation (0.94 Pearson) between the width of the confidence intervals and mean intensity (Supplementary Figure S2A). Therefore, we then normalized the width of the confidence intervals to the mean intensity values. The normalized confidence interval width (NCIW) appears to be a convenient metric as it is similar for all peaks, weakly dependent on their intensity. However, for the strongest peaks (peak intensity > 10) the NCIW is limited by 0.2, while for the weaker ones by 0.28 (Supplementary Figure S2B). Then we convert NCIW upper limits to the minimum confident fold changes by the following rule: min_fold = (1+NCIW/2)/(1-NCIW/2). Thus, we conclude that for the stronger peaks minimum confident fold change (*P*-value < 0.05) is ∼1.23, while for the weaker peaks it is ∼1.33.

### RNA-Seq — sample preparation

For RNA sequencing, *C. glutamicum* wild-type and the Δ*hrrA* mutant strain were cultivated under the same conditions as described for ChAP Sequencing. Both strains did not contain any plasmids and, hence, were cultivated without addition of antibiotics in biological duplicates. After 0 (no heme), 0.5 and 4 h, cells corresponding to an OD_600_ of 3 in 0.1 l were harvested in falcon tubes filled with ice by centrifugation at 4°C and 5000 × *g* for 10 min and the pellets were stored at −80°C. For the preparation of the RNA, the pellets were resuspended in 800 μl RTL buffer (QIAGEN GmbH, Hilden, Germany) and the cells disrupted by 3 × 30 s silica bead beating, 6000 rt/min (Precellys 24, VWR International GmbH, Darmstadt, Germany). After ultra-centrifugation (150 000 × *g*, 4°C, 1 h), the RNA was purified using the RNeasy Mini Kit (QIAGEN GmbH, Hilden, Germany) according to the supplier's manual. Subsequently, the ribosomal RNA was removed by running twice the workflow of the Ribo-Zero rRNA Removal Kit [Bacteria] (Illumina, CA, USA) in succession. Between steps, the depletion of rRNA as well as the mRNA quality was analysed using the TapeStation 4200 (Agilent Technologies Inc, Santa Clara, USA). After removal of rRNA, the fragmentation of RNA, cDNA strand synthesis and indexing was carried out using the TruSeq Stranded mRNA Library Prep Kit (Illumina, CA, USA) according to the supplier's manual. Afterward, the cDNA was purified using Agencourt AMPure XP magnetic beads (Beckman Coulter, IN, USA). The resulting libraries were quantified using the KAPA library quant kit (Peqlab, Bonn, Germany) and normalized for pooling. Pooled libraries were sequenced on a MiSeq (Illumina, CA, USA) generating paired-end reads with a length of 2  ×  75 bases. Data analysis and base calling were performed with the Illumina instrument software and stored as fastq output files.

### RNA-Seq — analysis

Sequencing reads quality was explored with the FastQC (https://www.bioinformatics.babraham.ac.uk/projects/fastqc/) tool. Since reads appeared to be of a good quality and did not harbor significant fraction of adapters or over-represented sequences, no pre-processing was undertaken. Identical reads were collapsed with a custom script in order to prevent gene levels’ misquantification caused by PCR overamplification. Reads were mapped to the *C. glutamicum* genome (NC_003450.3) with Bowtie2 (45). Bowtie2 was run with the following parameters: bowtie2 -1 [path to the reads, 1st mate] -2 [path to the reads, 2nd mate] -S [path to the mappings] –phred33 –sensitive-local –local –score-min C,90 –rdg 9,5 –rfg 9,5 -a –no-unal -I 40 -X 400 –no-mixed –ignore-quals.

The reads mapped to multiple locations were split proportionally between parental genes. That is, if 3 reads are mapped to gene A and gene B, expression of gene A is 10 and expression of gene B is 5, then 2 reads will go to gene A and 1 read to gene B. For each *C. glutamicum* gene (46) we assigned an expression value equal to the average read coverage over the gene region. These expression values were then normalized to TPM (transcripts per million) values (47).

Furthermore, we analyzed which genes are significantly differentially expressed between conditions. We set combinatorial thresholds on normalized GEC (gene expression change) [|expr1-expr2|/(expr1+expr2)] and MGE (mean gene expression) [log2((expr1+expr2)/2)] where ‘expr1’ is gene expression for the first condition and ‘expr2’ for the second. Thresholds were set in a way to achieve maximal sensitivity while keeping FDR < 0.05. FDR was estimated as GECintra/(GECintra + GECinter); where GECintra is a number of genes passed the thresholds based on intrasample GEC (that is, gene expression change between the replicates for the same condition), GECinter is a number of genes passed the thresholds based on intersample GEC (that is, gene expression change between two different conditions). Threshold function for GEC was defined as: 1 | if MGE < C; 2**(-A*MGE) + B | if MGE ≥ C; where A, B, C are parameters to be adjusted. Parameters A, B, C were adjusted with genetic algorithm optimization approach to achieve maximal sensitivity in discovery of differentially expressed genes while keeping FDR below    0.05.

### Measurement of cell-associated hemin

*C. glutamicum* was cultivated in 4 μM hemin as described above (see ChAP-Seq). To measure the cell-associated heme pool, CGXII minimal medium supplemented with 2% (w/v) glucose and 4 μM heme was inoculated to an OD_600_ of 3.5. Samples were taken 0.5, 2, 4, 9 and 24 h after addition of heme. Cells were harvested, resuspended in 100 mM Tris–HCl (pH 8) and adjusted to an OD_600_ of 100. Cells cultivated in 4 μM FeSO_4_ supplemented medium were taken as a control and harvested at the same time points. Absolute spectra of cells reduced with a spatula tip of sodium dithionite were measured at room temperature using the Jasco V560 with a silicon photodiode detector in combination with 5 mm light path cuvettes. Absorption values at 406 nm were normalized by subtracting the measured absorption values of Fe-cultivated cells.

### Electrophoretic mobility shift assays (EMSA)

The promotor regions of HrrA target genes (100 bp) were chosen based on the ChAP-Seq analyses and covered the maximal HrrA peak area (for primers see Supplementary Table S2). For quantitative measurements, Cy3-labeled oligonucleotides were used for the generation of the DNA fragments. Before addition of the DNA, HrrA was phosphorylated by incubation for 60 min with MBP-HrrSΔ1-248 in a ratio of 2:1 and 5 mM adenosine triphosphate. Binding assays were performed in a total volume of 20 μl using 15 nM DNA and increasing HrrA concentrations (75 and 375 nM) for the qualitative analyses and 10 nM DNA with increasing HrrA concentrations from 5–1000 nM for quantitative analyses, respectively. The binding buffer contained 20 mM Tris–HCl (pH 7.5), 50 mM KCl, 10 mM MgCl2, 5% (v/v) glycerol, 0.5 mM EDTA and 0.005% (w/v) Triton X-100. After incubation for 20 min at room temperature, the reaction mixtures were loaded onto a 10 % native polyacrylamide gel and subsequently separated and documented using a Typhoon TrioTM scanner (GE healthcare). The band intensities of unbound DNA were quantified using Image Studio Lite (Licor, Bad Homburg, Germany). The band intensities were normalized to the lane containing no DNA and plotted against the HrrA concentration in log_10_ scale. Apparent *K*_d_ values were calculated based on at least three gels each using a sigmoidal fit and the software GraphPad Prism 8. For the sigmoidal fit, *Y* = 0 and *Y* = 1 were set as top and bottom constraints. The turning point of the curve was defined as the apparent *K*_d_.

### TMPD oxidase assay

*C. glutamicum* wild-type strain and the Δ*hrrA* mutant were cultivated to an OD_600_ of 4 in CGXII minimal with or without the addition of 4 μM hemin. Subsequently, cells were disrupted in a Precellys^®^ homogenisator (VWR International GmbH, Darmstadt, Germany) using zirconia/silica-beads (Ø 0.1 mm, Roth, Karlsruhe) in 100 mM Tris–HCl (pH 7.5) buffer. Ultracentrifugation at 200 000 × *g* for 1 h was used for membrane isolation. The pellet was resuspended in 100 mM Tris×HCl buffer and the protein concentration was determined using a BCA assay. The N,N,N’,N’-Tetramethyl-*p*-phenylenediamine (TMPD) oxidase activity in the membrane faction was measured spectrophotometrically at 562 nm in a TECAN Reader (Thermo Fisher Scientific, Massachusetts, US) by injecting 200 μM TMPD (37). An extinction coefficient of 10.5 mM^−1^ cm^−1^ was used (48). One unit of activity was defined as 1 μmol of TMPD oxidized per minute. As a control for autooxidation, a sample containing only 100 mM Tris–HCl buffer was recorded after TMPD addition and substracted from the actual rates. Significance was evaluated by an unpaired t-test with a 95% confidence interval.

## RESULTS

### Genome-wide profiling of HrrA promoter occupancy

In previous studies, a number of direct HrrA target operons were described in *C. glutamicum* and *C. diphtheriae*, suggesting an important role of the HrrSA TCS in the control of heme homeostasis (17–20). It has to be noted, that the membrane embedded HrrS sensor kinase is also activated by endogenously synthesized heme (21) and that the addition of external heme leads to a boost of the HrrSA response. In this study, we investigated the genome-wide binding profile of HrrA using chromatin affinity purification of twin-Strep-tagged HrrA combined with DNA sequencing (ChAP-Seq). Importanty, qPCR experiments confirmed wild-type level expression of the twin-Strep-tagged version of HrrA.

**Figure 1.**
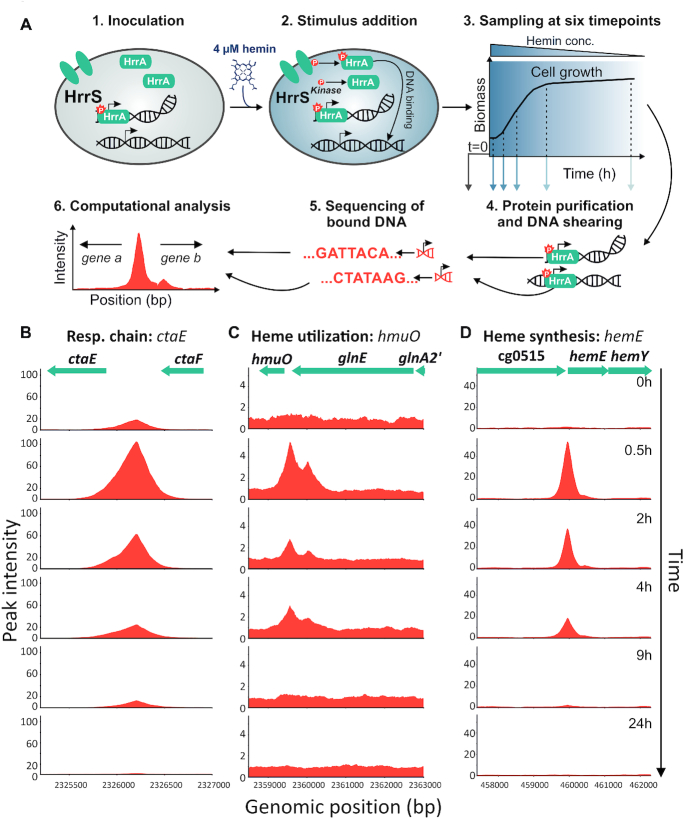
Genome-wide profiling of HrrA binding in response to addition of external heme. (**A**) ChAP-Seq analysis of the *Corynebacterium glutamicum* strain ATCC 13032::*hrrA*-C-twinstrep grown in iron-depleted glucose minimal medium before and after addition of 4 μM hemin. The experimental approach is briefly depicted: cells were harvested at the indicated time points, twin-Strep tagged HrrA was purified and co-purified DNA was sequenced to identify HrrA genomic targets. This approach resulted in the identification of more than 200 genomic regions bound by HrrA upon addition of hemin after 30 min. Exemplarily shown is the HrrA binding to regions upstream of operons involved in (**B**) the respiratory chain (*ctaE*), (**C**) heme degradation (*hmuO*) and (**D**) heme biosynthesis (*hemE*).

To obtain insights into the stimulus-dependent DNA association and dissociation, *C. glutamicum* cells were grown in iron-depleted glucose minimal medium, and samples were obtained before (T_0_) and 0.5, 2, 4, 9 and 24 h after the addition of 4 μM hemin. HrrA was purified, and the bound DNA fragments were sequenced (Figure 1A). We obtained substantial enrichment of known HrrA targets in response to heme (e.g. after 0.5 h: 5-fold *hmuO*, 54-fold *hemE*, 105-fold *ctaE*; Figure 1B–D, respectively) and identified more than 200 previously unknown HrrA-bound regions in the *C. glutamicum* genome (Supplementary Table S3).

**Figure 2.**
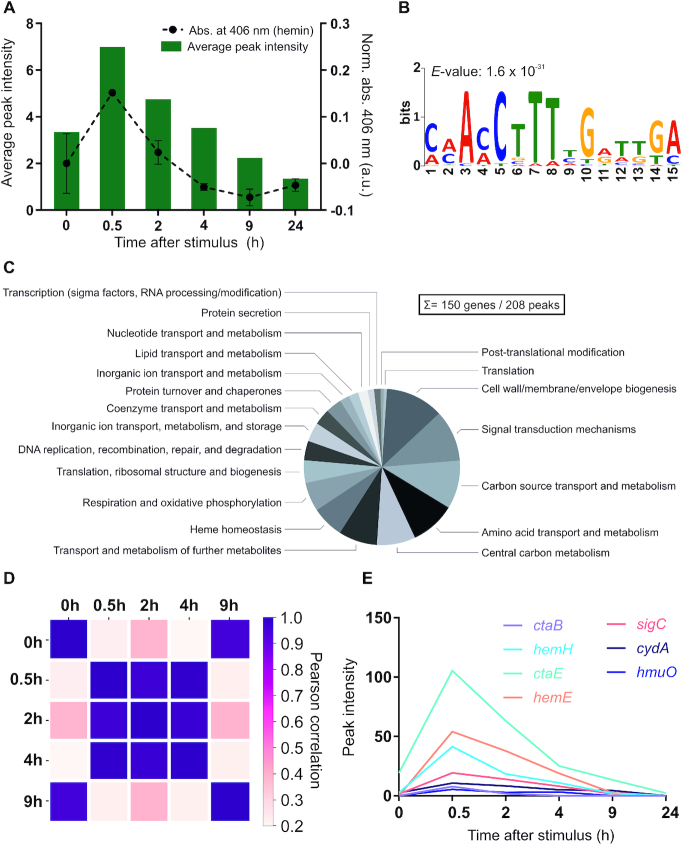
ChAP-Seq analysis revealed HrrA as a global regulator of heme homeostasis in *Corynebacterium glutamicum*. (**A**) HrrA binding in response to the addition of hemin. The bar plot reflects the average peak intensities among detected peaks in ChAP-Seq experiments (<800 bp to the next TSS). The binding was correlated with the amount of cell-associated hemin (dashed line), measured at corresponding time points by spectroscopy as described in ‘Materials and Methods’ section. (**B**) A binding motif was deduced from the sequences of the top 25 peaks (T_0.5_) using MEME v.5 analysis (http://meme-suite.org). (**C**) Pie chart presenting HrrA targets, which can be attributed to known functional categories (total of 272 genes, among which 128 encode proteins of unknown function, e.g. target genes within the CGP3 prophage region were excluded). For a complete overview of HrrA targets, see Supplementary Table S3. (**D**) Proportional behavior of the HrrA regulon. For each peak that passed the threshold (distance of <800 bp to the closest downstream or <200 to the closest upstream TSS) at time point A, the highest peak in the same region (±50 nt from the center of the peak) was selected for time point B and *vice versa*. Thus, ‘paired’ peaks for these two time points were obtained, and the Pearson correlation of the intensities of all paired peaks was calculated for all six time points. (**E**) Peak intensities of selected HrrA targets over time, as identified by ChAP-Seq.

As expected, the highest number of peaks was identified at the first time point after the heme pulse (0.5 h), with 199 peaks meeting our applied threshold (distance of <800 bp to the closest downstream or <200 bp to the closest upstream transcription start site (TSS)). In comparison, only 15 peaks showed a more than 2-fold enrichment before hemin addition (T_0_, Supplementary Table S3 and Figure S3). It has to be noted, that these 15 peaks detected at T_0_ appear to be specific HrrA targets, since none of them was detected in an input control sample. Overall, these data illustrate the fast and transient DNA binding by HrrA in response to heme. In general, the majority of the discovered HrrA binding sites were close to TSSs (Supplementary Figure S4). The binding of HrrA to 11 selected targets was confirmed by electrophoretic mobility shift assays (Supplementary Figure S5), and a palindromic binding motif was deduced (Figure 2B and Supplementary Figure S6).

The HrrA binding patterns depicted in Figure 1B–D are representative of many bound regions. Thirty minutes after the heme pulse, the average peak intensities increased ∼2.5-fold in comparison to those at T_0_ (Figure 2A). After 2 h of cultivation in hemin, the average peak intensity is declining and is, after 9 h, already below the starting level at T_0_ reaching a minimum in stationary phase (24 h). This is likely the result of the pre-cultivation and main cultivation under iron starvation conditions leading to a lowered intracellular heme pool. The dissociation of HrrA from its target promoters is, consequently, caused by rapid depletion of heme and a switch of HrrS from kinase to phosphatase state (23). Heme depletion was confirmed by spectroscopy of *C. glutamicum* cells (Figure 2A, dashed line) and was also obvious upon visual inspection (Supplementary Figure S7). Of all peaks, that passed our threshold, 128 were upstream of genes encoding hypothetical proteins, while 150 could be assigned to genes with known or predicted function (Figure 2C). Furthermore, we assessed the significance levels of HrrA binding changes between samples from different conditions or/and time-points. It turned out, that for the stronger peaks (peak intensity > 10) the minimum significant fold change (*P*-value < 0.05) is ∼1.23, while for the weaker peaks (peak intensity < 10) it is ∼1.33 (see ‘Materials and Methods’ section).

To analyze the synchronicity in the HrrA regulon, peak intensities were correlated over time. A relatively high correlation between peak intensities for the time points 0.5, 2 and 4 h (Figure 2D) showed that the system reacted proportionally for a majority of the binding sites and the strength of HrrA binding changed in response to heme availability. Relaxation of the system was observed after 9 h where peak intensities correlated well with T_0_.

### The HrrSA TCS coordinates heme homeostasis by integrating the response to oxidative stress and cell envelope remodeling

Our dataset confirmed the binding of HrrA to all previously known targets, including genes encoding components of heme biosynthesis (*hemE*, *hemH* and *hemA*), degradation (*hmuO*) and export (*hrtBA*) pathways and heme-containing complexes of the respiratory chain (*ctaE*-*qcrCAB* operon and *ctaD*). A comprehensive overview of all identified HrrA targets is presented in Supplementary Table S3; selected target genes are listed in Table 1. Among the more than 180 novel targets identified in this study, we observed HrrA binding upstream of *ctaB*, which encodes a protoheme IX farnesyltransferase that catalyzes the conversion of heme *b* to heme *o* (26) and upstream of *ctaC*, which encodes subunit 2 of the cytochrome *aa*_3_ oxidase. Remarkably, HrrA binding was also observed upstream of the *cydABDC* operon, which encodes the cytochrome *bd* oxidase of the respiratory chain. Altogether, this set of target genes highlights the global role of the HrrSA system in heme-dependent coordination of both branches of the respiratory chain. The HrrA regulon appeared to cover also the aspect of cofactor supply for the respiratory chain, as several HrrA targets encode enzymes involved in menaquinone reduction (*sdhCD*, *lldD* and *dld*).

**Table 1.** Selected target genes of HrrA

Locus tag	Gene name	Annotation	Dist. TSS^a^	Peak intensity^b^	log_2_(Δ*hrrA*/wt)^c^ T 0.5 h	log_2_(Δ*hrrA*/wt)^c^ T 4 h
**Heme homeostasis/metabolism**						
cg2445	***hmuO***	Heme oxygenase	43	5.4	−3.1	−3.8
cg0516	***hemE***	Uroporphyrinogen decarboxylase	17	54	3.1	2.2
cg0497	***hemA***	Glutamyl-tRNA reductase	−162	13	0.7	1.0
cg0517	***hemY***	Protoporphyrinogen oxidase	429	3.0	2.8	1.6
cg2079	***hemQ***	Putative chlorite dismutase-family protein, conserved		19	2.8	1.8
cg3156	***htaD***	Secreted heme transport-associated protein	−108	15	−0.3	−1.1
cg1734	***hemH***	Ferrochelatase	21	41	4.0	2.2
cg3247	***hrrA***	Heme-dependent response regulator	108	3.7	n.d.	n.d.
cg2201	***chrS***	Heme-dependent histidine kinase (*chrSA* operon)	32	2.5	−0.4	1.3
cg2202	***hrtB***	Heme exporter (*hrtBA* operon)	78	2.5	−1.0	4.3
**Respiratory chain**						
cg2406	***ctaE***	Cytochrome *aa*_3_ oxidase, subunit 3	307	105	−1.7	−0.8
cg2780	***ctaD***	Cytochrome *aa*_3_ oxidase, subunit 1	197	36	−1.1	−0.9
cg1301	***cydA***	Cytochrome *bd* oxidase	192	11	−0.7	−2.6
cg2409	***ctaC***	Cytochrome *aa*3 oxidase, subunit 2	47	22	−1.4	−1.0
cg1773	***ctaB***	Protoheme IX farnesyltransferase	667	7.9	0.4	−1.4
cg0445	***sdhC***	Succinate:menaquinone oxidoreductase, cytochrome b subunit	83	38	−1.7	−1.6
cg3226		L-lactate permease, operon with *lldD*	533	5.5	−1.7	0.9
**Glucose uptake**						
cg2121	***ptsH***	Phosphocarrier protein HPr, general component of PTS	−70	2.1	−1.2	−0.3
cg1537	***ptsG***	Glucose-specific EIIABC component EIIGlc of PTS	70	1.6	−1.1	−0.1
cg2091	***ppgG***	Polyphosphate glucokinase	199	266	0.2	−0.8
cg0223	***iolT1***	Myo-Inositol transporter 1, alternative glucose uptake system	73	2.0	−1.0	−0.7
**Signal transduction**						
cg0986	***amtR***	Master regulator of nitrogen control, repressor, TetR-family	366	1.8	0.3	0.1
Cg2461	***benR***	Transcriptional regulator, LuxR-family	229	5.6	−0.1	−1.2
cg2761	***cpdA***	cAMP phosphodiesterase	309	4.2	0.4	−0.5
cg0309	***sigC***	Extracytoplasmid-function σ factor, control of branched quinol oxidation pathway	29	19	2.1	0.6
cg0444	***ramB***	Transcriptional regulator, involved in acetate metabolism	83	38	−0.7	−0.6
cg2831	***ramA***	Transcriptional regulator, acetate metabolism, LuxR-family	−10	2.1	−0.5	0.6
**Oxidative stress**						
cg0310	***katA***	Catalase	132	19	−0.7	−1.2
cg0831	***tusG***	Trehalose uptake system, ABC-type, permease protein	−30	1.8	0.0	−0.2
cg1791	***gapA***	Glyceraldehyde-3-phos. dehydrogenase, glycolysis	86	3.9	−0.3	−0.4
cg1069	***gapB***	Glyceraldehyde-3-phos. dehydrogenase, gluconeogenesis	175	2.4	1.6	−0.1
**Cell envelope**						
cg2077	***aftC***	arabinofuranosyltransferase	271	3.0	−0.3	−0.2
cg3323	***ino1***	D-myo-inositol-1-phosphate synthase	−46	4.2	1.7	0.6
cg0337	***whcA***	WhiB homolog, role in SigH-mediated oxidative stress response	−21	2.1	−0.5	−0.7
cg0306	***lysC***	Aspartate kinase	32	13	0.7	0.1
cg0422	***murA***	UDP-N-acetylenolpyruvoylglucosamine reductase	591	3.5	−0.3	−0.1

^a^Distance of the HrrA binding peak, identified via ChAP-Seq, to the start codon (TSS).

^b^The corresponding peak intensity.

^c^Relative ratio of the transcript levels of the Δ*hrrA* deletion mutant compared to the wild-type (log_2_ fold change). The values are derived from a comparison between the two strains 0.5 and 4 h after hemin addition. The log_2_(Δ*hrrA*/wt) value for was not determined for the deleted *hrrA* gene (n.d.).

This table summarizes results from the HrrA ChAP-Seq analysis of the *C*. *glutamicum* strain ATCC 13032::hrrA-C-twinstrep and the transcriptome analysis of *C. glutamicum* wild-type and strain ΔhrrA (complete datasets are provided in Supplementary Tables S3 and 4, respectively). For both experiments, cells were grown on glucose minimal medium and 4 M heme (see ‘Materials and Methods’ section).

Besides the known heme biosynthesis targets (*hemE*, *hemH* and *hemA*), HrrA binding was also observed upstream of a gene (*hemQ*) encoding a putative dismutase-family protein. However, in actinobacteria, it was proposed that these proteins do not possess chlorite dismutase activity but instead are essential for heme synthesis (49). Furthermore, we observed binding of HrrA upstream of the *chrSA* operon encoding the second TCS involved in heme-dependent regulation in *C. glutamicum*. This finding therefore confirmed the previously postulated cross-regulation of these TCS at the level of transcription (24,50).

Furthermore, HrrA binding was also observed upstream of several genes involved in the oxidative stress response, including *katA*, encoding catalase, *tusG*, encoding a trehalose uptake system (51), and upstream of *gapA* and *gapB* (glyceraldehyde-3-phosphate dehydrogenase, glycolytic and gluconeogenetic, respectively) (52,53). In line with these findings, the phenotypic analysis of a *C. glutamicum hrrA* mutant revealed a significantly higher sensitivity to oxidative stress (treatment with H_2_O_2_) in comparison to the wild-type (Supplementary Figure S8). These findings suggest that the HrrSA system not only controls heme biosynthesis and degradation but also integrates the response to heme-induced oxidative stress.

A further important class of HrrA targets is represented by genes associated with the regulation or maintenance of the *C. glutamicum* cell envelope. The gene products of these previously unknown HrrA targets are, for instance, involved in the synthesis of peptidoglycan (*murA*), the peptidoglycan precursor meso-2,6-diaminopimelate (mDAP), inositol-derived lipids (*ino1*) and arabinogalactan (*aftC*). Furthermore, HrrA binding was revealed upstream of a number of genes encoding global transcriptional regulators (e.g. *ramA*, *ramB* and *amtR*), adding a further level of complexity to this systemic response to heme.

### Temporal dynamics of promoter occupancy reveal hierarchy in the HrrA regulon

With the time-resolved and genome-wide analysis of HrrA binding, we were also able to visualize distinct binding patterns of HrrA in response to addition and depletion of heme. Consequently, we asked whether the binding patterns (ChAP-Seq coverage) could provide information regarding the apparent dissociation constant (*K*_d_) of HrrA to specific genomic targets. We compared the *in vivo* binding patterns of HrrA to *ctaE*, *hmuO* and *cydAB* (Figure 1 ,2E and Supplementary Table S3). While a comparably high peak was observed upstream of the *ctaE* promoter—even before the addition of heme (T_0_)—the binding of HrrA to the promoter of *hmuO* occurred with apparently high stimulus dependency and appeared to be rather transient, as HrrA was fully dissociated from this promoter 9 h after the addition of hemin (Figure 1B and C).

Subsequently, we determined the *in vitro* affinity of phosphorylated HrrA to the promoter regions of *ctaE*, *cydAB* and *hmuO* (Table 2 and Supplementary Figure S9). Consistent with the ChAP-Seq data, we measured the highest affinity of HrrA to P*_ctaE_*with an apparent *K*_d_ of 125 nM. We therefore hypothesize that the *ctaE* promoter is a prime target that is constitutively activated by HrrA (Supplementary Table S3) to maintain high gene expression of the operon encoding the *bc*_1_–*aa*_3_ supercomplex. In line with this hypothesis, we also found a high HrrA binding peak upstream of the other operons encoding components of the *bc*_1_–*aa*_3_ supercomplex (*ctaD* and *ctaCF*, Table 1 and Supplementary Table S3).

**Table 2.** Apparent *K*_d_values of HrrA to the promoters of *hmuO*, *ctaE*, *sigC* and *cydA*

Promoter	Function	Apparent *K*_d_ value (nM)	95% confidence interval (nM)	R²	Peak intensity after hemin addition (ChAP-Seq)
P*_hmuO_*	Heme oxygenase	196	182–212	0.95	10
P*_ctaE_*	Cytochrome *aa*_3_ oxidase	125	117–132	0.97	53
P*_sigC_*	ECF sigma factor σ^C^	271	247–299	0.96	25
P*_cydA_*	Cytochrome *bd* oxidase	350	318–386	0.96	18

The affinity of phosphorylated HrrA to the indicated regions was measured using purified protein in increasing concentrations and its ability to shift 10 nM DNA fragments of ∼100 bp size covering the maximal ChAP-Seq peak (for detailed information, see Supplementary Figure S9).

In contrast, we measured an almost 3-fold higher apparent *K*_d_ (350 nM) for P*_cydAB_*, which was consistent with the relatively transient binding pattern observed for this target. With an apparent *K*_d_ of 196 nM, the *in vitro* binding affinity of HrrA to the *hmuO* promoter was rather high considering the genomic coverage measured in the ChAP-Seq analysis. However, *in vitro* analysis does not account for the widespread interference among regulatory networks *in vivo*. In the particular example of *hmuO*, the pattern of HrrA binding was likely the result of interference with the global regulator of iron homeostasis, DtxR, which has previously been described to repress *hmuO* expression by binding to adjacent sites (54). Taken together, these results suggest that *in vivo* promoter occupancy is not only influenced by the binding affinity of the regulator to the particular target, but also significantly shaped by network interference. Consequently, high *in vivo* promoter occupancy indicates high binding affinity, but conclusions based on weakly bound regions may be confounded by competition with other binding factors.

### HrrA activates the expression of genes encoding components of both branches of the quinol oxidation pathway

To evaluate how HrrA binding affects the expression of individual target genes, we analyzed the transcriptome (RNA-Seq) of the *C. glutamicum* wild-type strain (ATCC 13032) as well as a Δ*hrrA* mutant (Supplementary Table S4). Analogous to the ChAP-Seq experiments, RNA-Seq analysis was performed prior to the addition of heme (T_0_) as well as 0.5 and 4 h after the heme pulse (in medium containing no other iron source). The RNA-Seq analysis was performed in two independent biological replicates (for inter-replicate variation, see Supplementary Table S5).

**Figure 3.**
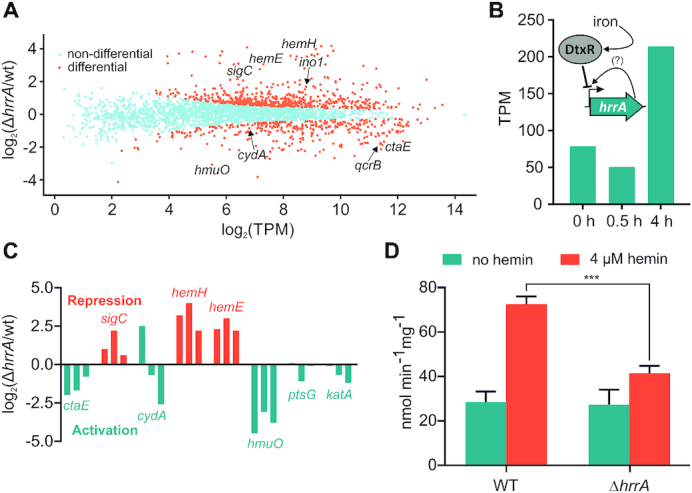
Differential gene expression analysis of wild-type *Corynebacterium glutamicum* and a Δ*hrrA* mutant. (**A**) Differential gene expression analysis (RNA-Seq) revealed 120 upregulated and 154 downregulated genes in the *hrrA* deletion strain compared to the wild-type (in transcripts per million, TPM) after 30 min of cultivation in iron-depleted glucose minimal medium containing 4 μM heme. (**B**) Expression levels of *hrrA* (TPM) 0, 0.5 and 4 h after the addition of hemin. A scheme depicts HrrA autoregulation and iron-dependent DtxR repression (24). (**C**) Impact of *hrrA* deletion on the transcript levels of six selected target genes at three different time points (0, 0.5, 4 h; orange: HrrA acts as a repressor, turquoise: HrrA acts as an activator). (**D**) Measurement of cytochrome *aa*_3_ oxidase activity using the TMPD oxidase assay in *C. glutamicum* wild-type and Δ*hrrA* grown with or without 4 μM heme.

At T_0_, before the addition of heme, already 212 genes showed a more that 2-fold altered expression level in Δ*hrrA* cells compared to wild-type cells (Δ*hrrA*/wt). Directly after the addition of heme (0.5 h), the expression of 309 genes changed more than 2-fold. (Supplementary Table S4 and Figure 3A, orange dots). Of these genes, 174 were upregulated and 135 were downregulated in the *hrrA* deletion strain. Four hours after addition of heme, only 167 genes exhibited a >2-fold increase or decrease (scatter plots for additional time points are presented in Supplementary Figure S10).

The *hrrA* expression decreased after 0.5 h upon the addition of heme, which was likely caused by DtxR repression in response to increased intracellular iron levels (Figure 3B) (24). In contrast, after 4 h of cultivation, *hrrA* levels significantly increased, reflecting the depletion of heme as an alternative iron source and dissociation of DtxR. Furthermore, differential gene expression analysis revealed HrrA to be an activator of all genes encoding components of the respiratory chain (*ctaE*, *ctaD*, *ctaF* and *cydAB*) and as a repressor of heme biosynthesis (*hemA*, *hemE* and *hemH)* (Figure 3C). The impact on the cytochrome *bc*_1_–*aa*_3_ supercomplex was also confirmed by measuring the activity of the *aa*_3_ oxidase, which was about 2-fold reduced in a *hrrA* mutant in comparison to the wild-type when grown on heme (Figure 3D). Additionally, expression of *lldD* (L-lactate dehydrogenase) as well as *sdhCD* (succinate dehydrogenase) contributing to the reduced menaquinone pool was downregulated more than 3-fold upon deletion of *hrrA*. In addition to these considerable differences between the wild-type and the Δ*hrrA* mutant, we also observed decreased mRNA levels of genes involved in the oxidative stress response (e.g. *katA*) or cell envelope remodeling (e.g. *murA*) in the Δ*hrrA* mutant, suggesting HrrA to be an activator of these targets.

In some cases, promoter occupancy by HrrA did not result in altered expression levels of the particular target gene in a Δ*hrrA* mutant under the tested conditions (Table 1 and Supplementary Table S3). This finding is, however, not surprising considering the multiplicity of signals and regulators affecting gene expression. Under changing environmental conditions, transcription factor binding will not necessarily always be translated in an altered gene expression of the respective target. When we compare the results obtained from RNA-Seq and ChAP-Seq analysis, 269 genes out of 309 genes featuring an >2-fold change in gene expression did not show HrrA binding in their upstream promoter region. Looking at all HrrA targets (ChAP-Seq analysis) on a global scale, there is, nevertheless, a significantly higher impact on gene expression in a strain lacking *hrrA* for all targets bound by HrrA in comparison to non-targets (unbound, Supplementary Figure S11). Overall, 109 out of 228 HrrA targets featured a significantly altered gene expression in the *hrrA* mutant (64 increased and 55 decreased).

### HrrA determines the prioritization of terminal cytochrome oxidases by repression of *sigC*

**Figure 4.**
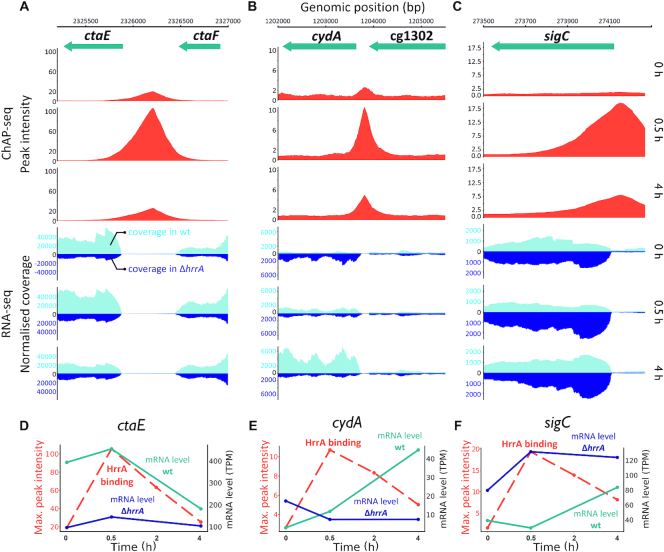
HrrA prioritizes the expression of genes encoding components of the *bc*_1_–*aa*_3_ supercomplex. Depicted are HrrA binding peaks as identified by ChAP-Seq analysis (Figures 1 and 2) in comparison to the normalized coverage of RNA-Seq results (wild-type and the Δ*hrrA* mutant) for the genomic loci of *ctaE* (**A** and**D**), *sigC* (**B** and**E**) and *cydA* (**C** and**F**). D–F: HrrA binding (max. peak intensities measured by ChAP-Seq experiments) and the mRNA levels (in transcripts per million, TPM) of the respective genes in the Δ*hrrA* strain as well as in wild-type *Corynebacterium glutamicum* cells 0, 0.5 and 4 h after the addition of hemin.

**Figure 5.**
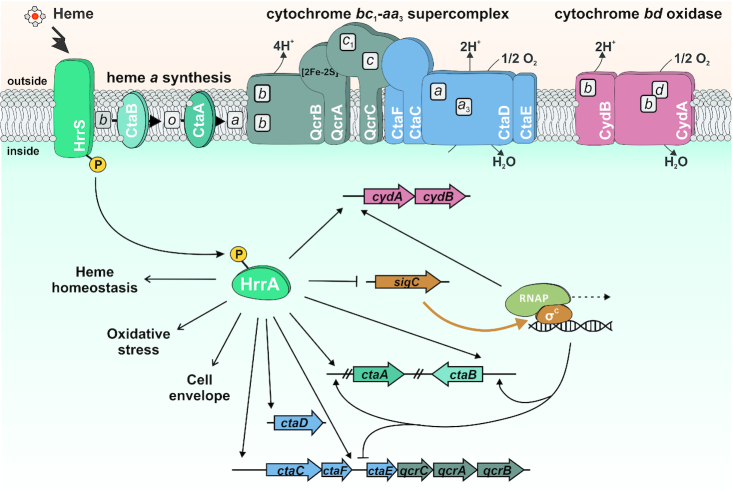
Model of heme-responsive control of components of the respiratory chain by HrrSA. The results of this study reveal HrrSA as a global regulator of heme homeostasis coordinating the expression of genes involved in heme biosynthesis, oxidative stress responses, glucose uptake and cell envelope remodeling. Genes encoding the components of the branched respiratory chain of *Corynebacterium glutamicum* comprise an important part of the HrrA regulon. While HrrA acts as an activator of almost all components (*ctaE-qcrCAB*, *ctaB*, *cydAB*), it represses transcription of the *sigC* gene encoding an important sigma factor required for *cydAB* expression. This regulatory network architecture consequently confers prioritization to the synthesis of the more efficient proton pump, the cytochrome *bc*_1_–*aa*_3_ supercomplex. Bordered boxes, b, c, a, d: heme *b*, heme *c*, heme *a*, heme *d*.

The results from ChAP-Seq and RNA-Seq experiments highlight the important role of HrrA in the control of the respiratory chain, including cofactor supply. Our data revealed that HrrA activates the expression of genes encoding the cytochrome *bc*_1_-*aa*_3_ supercomplex (*ctaE-qcrCAB*, *ctaD*, *ctaCF*) and of *cydAB*, encoding the cytochrome *bd* branch of the respiratory chain (Figure 4 and Supplementary Table S3). Remarkably, the mRNA profiles of the corresponding operons exhibited significantly delayed activation of *cydAB* in response to heme, which was abolished in the Δ*hrrA* mutant (Figure 4). In contrast, *ctaE* expression was significantly higher in wild-type cells, even before hemin addition (T_0_), but showed a further induction after stimulus addition (T 0.5 h, Supplementary Table S4). Notably, we also observed binding of HrrA upstream of *sigC*, encoding an ECF sigma factor that was shown to be involved in the activation of the *cydABDC* operon (35). The mRNA level of *sigC* increased more than 2-fold in the Δ*hrrA* mutant, indicating HrrA to be a repressor of this gene (Figure 4). Consistent with this hypothesis, *sigC* expression was slightly decreased in response to the addition of heme, which correlated with increased HrrA peak intensity (Figure 4F). Additionally, the higher *cydAB* expression, observed in the Δ*hrrA* strain before addition of stimulus (Figure 4B) is likely the effect of increased *sigC* expression (Figure 4C). Dissociation of HrrA from P*_sigC_* at a later time point (4 h after heme pulse) led to derepression of *sigC* coinciding with an increased expression of *cydAB* in the wild-type. Because *cydAB* levels were constitutively low in the Δ*hrrA* mutant in response to heme, we hypothesized that activation by HrrA together with an additional boost by SigC (Figure 5) leads to delayed activation of *cydAB* after the heme pulse. This regulation enables cells to channel most of the available heme pool into the more efficient cytochrome *bc*_1_–*aa*_3_ supercomplex. The lower apparent *K*_d_ of HrrA for the *ctaE* promoter (125 nM) compared to P*_cydAB_* (350 nM) or P*_sigC_* (270 μM) also reflects this prioritization of HrrA targets (Table 2). Consequently, this almost 3-fold decrease in affinity (apparent *K*_d_) increases the threshold for HrrSA activity to control these targets.

### HrrA activates PTS-dependent and -independent glucose uptake

Besides the activation of all components constituting the respiratory chain, ChAP-Seq experiments and transcriptome analysis revealed HrrA as a direct activator of genes encoding components of the phosphotransferase (PTS) system (*ptsH* and *ptsG*) and of *iolT1* encoding inositol permease with a reported function as a PTS-independent glucose uptake system (55). Remarkably, the gene *ppgK*, encoding the polyphosphate glucokinase was among the targets with the highest HrrA peak and showed reduced expression in the 4 h sample (Table 1 and Supplementary Table S4). These results emphasize that cellular respiration and glucose uptake is coordinated via the HrrSA system in response to cellular heme levels.

## DISCUSSION

In this work, we applied a genome-wide approach to study the ‘heme-responsive regulator’ HrrA in *C. glutamicum* and identified more than 200 genomic target regions of this response regulator. This intriguingly diverse set of target genes, encoding enzymes involved in heme biosynthesis, heme-containing proteins and components of the respiratory chain as well as proteins involved in oxidative stress response, glucose uptake and cell envelope remodeling, provided unprecedented insight into the systemic response to heme coordinated by the TCS HrrSA.

In Gram-positive bacteria, TCSs appear to play a central role in transient heme sensing, and heme-responsive systems have been described in several prominent pathogens, including *C. diphtheriae*, *S*. *aureus* and *B*. *anthracis* (15–18). However, for all prokaryotic heme regulatory systems, only a small number of target genes have been described to date, focusing on targets involved in degradation (*hmuO* (18,56)), heme export (*hrtBA* (19,57)) or heme biosynthesis (*hemA* (18,20)). Systems orthologous to HrrSA are present in almost all corynebacterial species and the high amino acid sequence identity shared by the response regulators (87%, between *C. glutamicum* and *C. diphtheriae* HrrA) suggests that the important role of HrrSA in the control of heme homeostasis is conserved. In many corynebacteria, including *C. diphtheriae*, control of heme homeostasis is shaped by the tight interplay of HrrSA with a second heme-dependent system, ChrSA. While the present study emphasized that HrrSA governs a large and complex homeostatic response, the only known target of the response regulator ChrA in *C. glutamicum* is the divergently located operon *hrtBA* encoding a heme export system. There is, however, also evidence for a cross-regulation between the TCSs, not only by cross-phosphorylation but also on the transcriptional level (23,24). In *C. diphtheria*, evidence for more overlap between the regulons of the TCSs has been provided, since both response regulators were shown to control a common set of target genes including *hrtBA*, *hemA* and *hmuO* (20,58). Genome-wide analysis of these systems have, however, not been performed so far and *invitro* protein–DNA interaction studies may not necessarily reflect the *invivo* promoter preferences of these highly similar systems.

### Coping with heme stress

While being an essential cofactor for many proteins, heme causes severe toxicity to cells at high levels (4). In mammalian cells, the BACH1 regulator is inactivated by heme binding and plays a key role in maintaining the balance of the cellular heme pool (8,59). Heme oxygenases are targets of various heme-dependent regulators (18,60–61), and consistent with this principle, the mammalian *HMOX1* gene, encoding an NADPH-dependent oxygenase, is regulated by BACH1 (59). Other identified BACH1 targets are involved in redox regulation, the cell cycle, and apoptosis as well as subcellular transport processes (9,62–63).

Although neither the regulator nor the constitution of the regulon is conserved, the responses of BACH1 and HrrSA share a similar logic. Analogous to eukaryotic BACH1, we observed HrrA-mediated activation of genes involved in the oxidative stress response, including *katA*, which appears to be required to counteract oxidative stress caused by elevated heme levels (Supplementary Figure S8).

Remarkably, HrrA binding was also observed upstream of both *gapA* and *gapB*, which encode glyceraldehyde-3-phosphate dehydrogenases (GapDHs) involved in glycolysis and gluconeogenesis, respectively. Previous studies in baker's yeast and mammalian cells have revealed that oxidative stress may block glycolysis by inhibiting GapDH (53,64). Furthermore, GapDH of *C. diphtheriae* was recently shown to be redox-controlled by S-mycothiolation (65). Slight activation of *gapA* by HrrA may thus counteract an impaired glycolytic flux under conditions of heme stress.

Furthermore, several HrrA targets play a role in the biosynthesis and remodeling of the corynebacterial cell envelope, including *ino1*, which is required for the synthesis of inositol-derived lipids (66), *lysC*, providing the peptidoglycan precursor meso-2,6-diaminopimelate (mDAP) and *murA* (Table 1). Taken together, these insights emphasize the important role of the HrrSA system in the control of heme stress responses.

### From networks to function

Genome-wide analysis of regulatory networks may provide important hints toward the physiological function of genes. An example is provided by the HrrA-dependent regulation of cg2079 (*hemQ*), described in this study (Table 1). In actinobacteria, it was recently proposed that these proteins inherit an essential role in heme biosynthesis (49,67). The finding that HrrA binds to the promoter of this gene and represses its expression supports a role of HemQ in heme biosynthesis in *C. glutamicum*. Among the direct targets of HrrA are many further targets encoding proteins of unknown function, including several ABC transport systems with a potential role in heme uptake or export. Therefore, this dataset provides guidance for further functional analysis of these HrrA targets to decipher their role in heme homeostasis.

### Coordinated control of the respiratory chain

Among the most significantly affected targets in the Δ*hrrA* mutant were many genes encoding components of the respiratory chain (26). These genes comprise all the genes constituting the cytochrome *bc*_1_-*aa*_3_ branch of the respiratory chain (*ctaE-qcrCAB*, *ctaCF* and *ctaD*) (68); genes encoding the cytochrome *bd* branch (*cydAB* (26)); *ctaA (**69**) and ctaB* (70), encoding enzymes responsible for heme *a* synthesis; and *lldD* and *dld*, encoding lactate dehydrogenases that contribute to the reduced menaquinone pool (26) (Figure 4; Supplementary Figure S12 and Table S3).

In a recent study, Toyoda and Inui described the ECF sigma factor σ^C^ to be an important regulator of both branches of the *C. glutamicum* respiratory chain. The *ctaE-qcrCAB* operon was shown to be significantly downregulated after σ^C^ overexpression due to binding of the sigma factor to the antisense strand of the promoter (35). Here, we demonstrated that this repression is counteracted by HrrA, which not only represses *sigC* but also activates *ctaE-qcrCAB* expression. While the two proteins have antagonistic effects on the expression of the supercomplex, both σ^C^ and HrrA positively regulate the *cyd* operon, encoding the cytochrome *bd* branch of the respiratory chain (Figure 5).

Interestingly, a hierarchy in the regulon was reflected by the differences in the apparent *K*_d_ values of HrrA with P*_cydA_* and P*_sigC_*, which were 2-fold lower than those with the promoter of *ctaE*. These findings were also consistent with the ChAP-Seq experiments, where the peaks upstream of *ctaE* and *ctaD* were among the highest peaks at T_0_ and after 0.5 h (Figure 4A). These data suggest that under conditions of sufficient heme supply, production of the cytochrome *bc*_1_–*aa*_3_ supercomplex is preferred, which is highly effective but requires the incorporation of six heme molecules (in contrast to only three molecules for the synthesis of the *bd* oxidase). Repression of *sigC* by HrrA and the relatively low affinity to the *cydAB* promoter results in delayed production of the *bd* branch. Under the applied aerobic conditions, available heme is thus first channeled to the cytochrome *bc*_1_–*aa*_3_ supercomplex before the cytochrome *bd* oxidase is used, which is less efficient but has a higher oxygen affinity. Remarkably, HrrA was also found to activate expression of genes involved in PTS-dependent (*ptsH* and *ptsG*) and -independent (*iolT1*) glucose uptake thereby ensuring a high glucose uptake rate under conditions of active cellular respiration.

### Interference with other regulatory networks

Deletion of the *hrrA* gene led to more than 2-fold upregulation of 174 genes, while 135 genes were downregulated after the addition of heme. Several other genes were significantly affected but to a lesser extent. Remarkably, among the direct target genes controlled by HrrA, we identified several prominent global regulators, including the regulators of acetate metabolism *ramA* and *ramB* (71,72), and *amtR* encoding the master regulator of nitrogen control (73). Furthermore, *cpdA* encoding a cAMP phosphodiesterase playing a key role in the control of cellular cAMP levels in *C. glutamicum* (74) was found to be under direct control of HrrA. These examples illustrate the profound influence of HrrA on cellular networks and the systemic response cells have programmed to respond to heme availability.

## CONCLUSION

Genome-wide analyses of targets controlled by prokaryotic transcription factors will change our view on many systems we believe to know. In this study, we provide an unprecedented insight into the systemic response to heme coordinated by the TCS HrrSA. Given the many properties of this molecule, the complexity of this response is actually not surprising but paves the way for further functional analysis of HrrA targets with so far unknown functions in heme homeostasis.

## DATA AVAILABILITY

The custom-developed software used in this study is publicly available at GitHub repository under the link https://github.com/afilipch/afp.

All sequencing data were deposited in the GEO database under the accession numbers GSE121962 (ChAP-Seq) and GSE120924 (RNA-Seq).

## Supplementary Material

gkaa415_Supplemental_Files
