# Supplementary material for: HrrSA orchestrates a systemic response to heme and determines prioritization of terminal cytochrome oxidase expression
Source: Nucleic Acids Res. 2020 May 26;48(12):6547–62. doi: 10.1093/nar/gkaa415 (PMC7337898; doi:10.1093/nar/gkaa415)
Supplement: gkaa415_Supplemental_Files [file gkaa415_supplemental_files.zip › 20200426_HrrA_Supplement.pdf]

Supplementary Information for

HrrSA orchestrates a systemic response to heme and determines prioritisation of terminal cytochrome oxidase expression

Marc Keppel[1#], Max Hünnefeld[1#], Andrei Filipchyk[1#], Ulrike Viets[1], Cedric-Farhad Davoudi[1], Aileen Krüger[1], Eugen Pfeifer[2], Christina Mack[1], Tino Polen[1], Meike Baumgart[1], Michael Bott[1], and Julia Frunzke[1*]

[1]Institute of Bio- und Geosciences, IBG-1: Biotechnology, Forschungszentrum Jülich, 52425 Jülich, Germany
[2]Microbial Evolutionary Genomics, Institute Pasteur, 75015 Paris, France

*Corresponding author:
Julia Frunzke; Email: j.frunzke@fz-juelich.de; Phone: +49 2461 615430

[#]These authors contributed equally to this work.

This PDF file includes:

Figure S1: Schematic overview of the convolution profiling.
Figure S2: Assessment of significance for the reported peak intensity values.
Figure S3: Global binding pattern of HrrA in the *C. glutamicum* genome in response to hemin addition.
Figure S4: Distribution of distances from HrrA binding peaks centers to the closest gene start site (transcription start site, TSS).
Figure S5: HrrA binding to selected target promotor regions.
Figure S6: Derivation of a HrrA binding motif revealed a weakly conserved palindromic sequence.
Figure S7: Visual inspection of *C. glutamicum* cells before and after addition of heme.
Figure S8: Growth assays revealed an increased sensitivity of Δ*hrrA* cells against oxidative stress.
Figure S9: Binding affinity of HrrA to selected target promoters.
Figure S10: Time-resolved differential gene expression analysis.
Figure S11: Correlation of HrrA binding and expression change.
Figure S12: HrrA coordinates expression of *ctaA* and *ctaB* in response to heme.

Table S1: Bacterial strains and plasmids used in this study.
Table S2: Oligonucleotides used in this study.
Table S5: Pearson correlation for the gene expression values (TPM) between the two biological replicates.

SI References

Other supplementary materials for this manuscript include the following separate files:

Table S3: Full Dataset, binding peaks HrrA and transcriptome analysis of *C. glutamicum* wild type and Δ*hrrA.*
Table S4: Filtered dataset of time resolved transcriptome analysis of *C. glutamicum* wild type and Δ*hrrA* with genes showing at least two-fold alteration.

Supplementary Figures

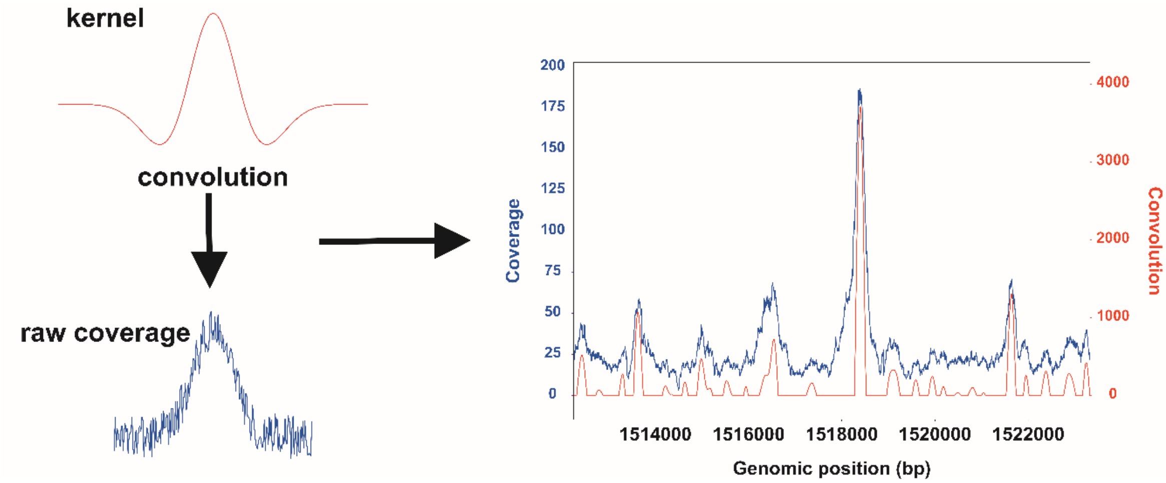

Figure S1: Schematic overview of the convolution profiling. Read coverage was convolved with negative second order Gaussian kernel. The convolved read coverage was then scanned to discover the local maxima (peaks).

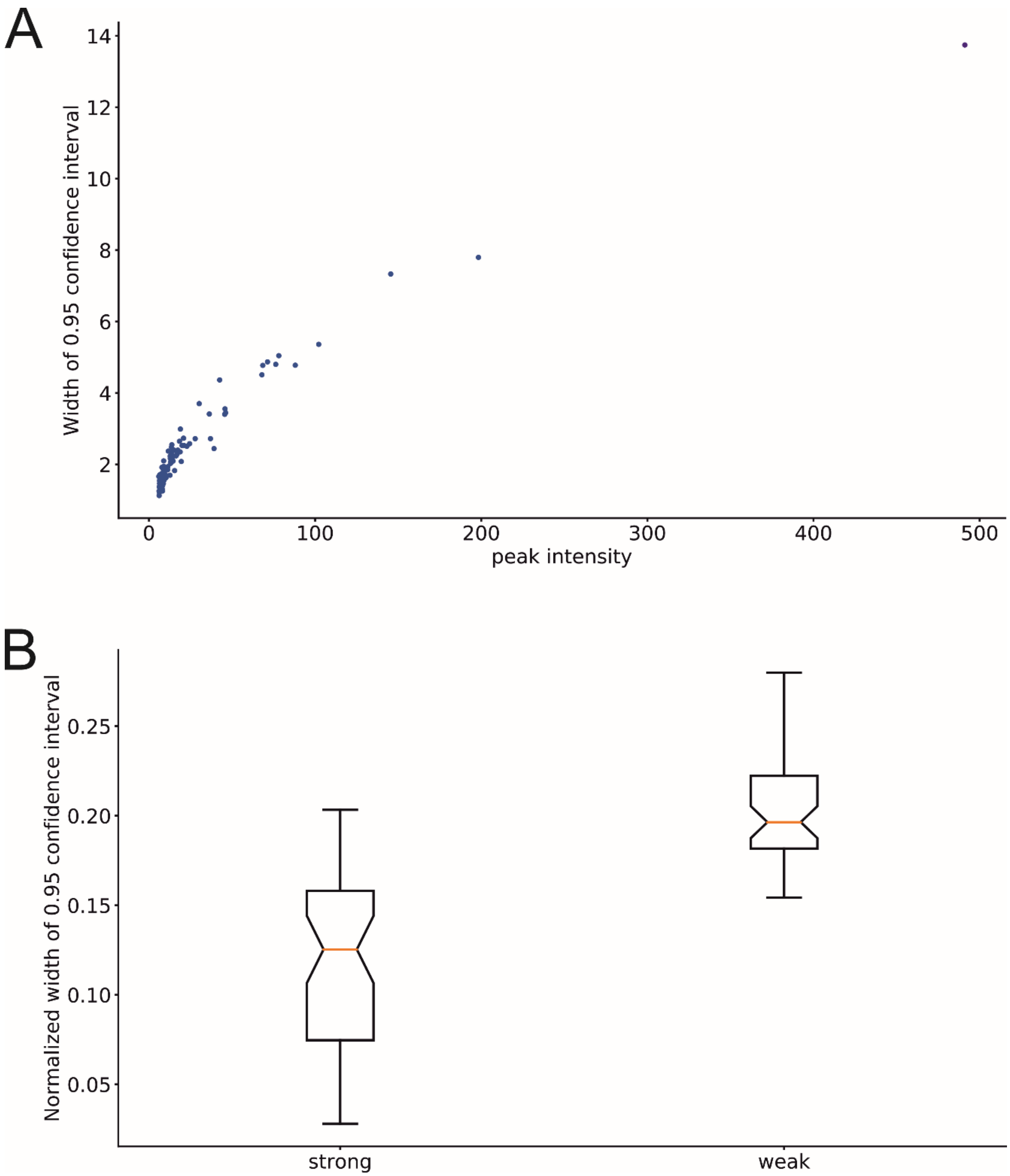

Figure S2: Assessment of significance for the reported peak intensity values. (A) The error of peak intensity value (0.95 confidence intervals width) linearly depends on its absolute value. The stronger the peak the less the confidence in its absolute value. In contrast, the relative error normalized to peak intensity is similar for peaks of various strength, hence can be used as universal measure for significance assessment. (B) Distribution of the normalized confidence intervals width (NCIW) among the detected peaks. The distribution is represented as box plots with box edges at 1st and 3rd quantiles and box whiskers at minimum and maximum values. For the weak peaks (peak intensity <10) average NCIW is around 0.2 and limited by 0.28, while for the strong ones the average NCIW is around 0.13 and limited by 0.2. The upper limits were taken for the final estimation, as the most conservative confidence evaluation was pursued.

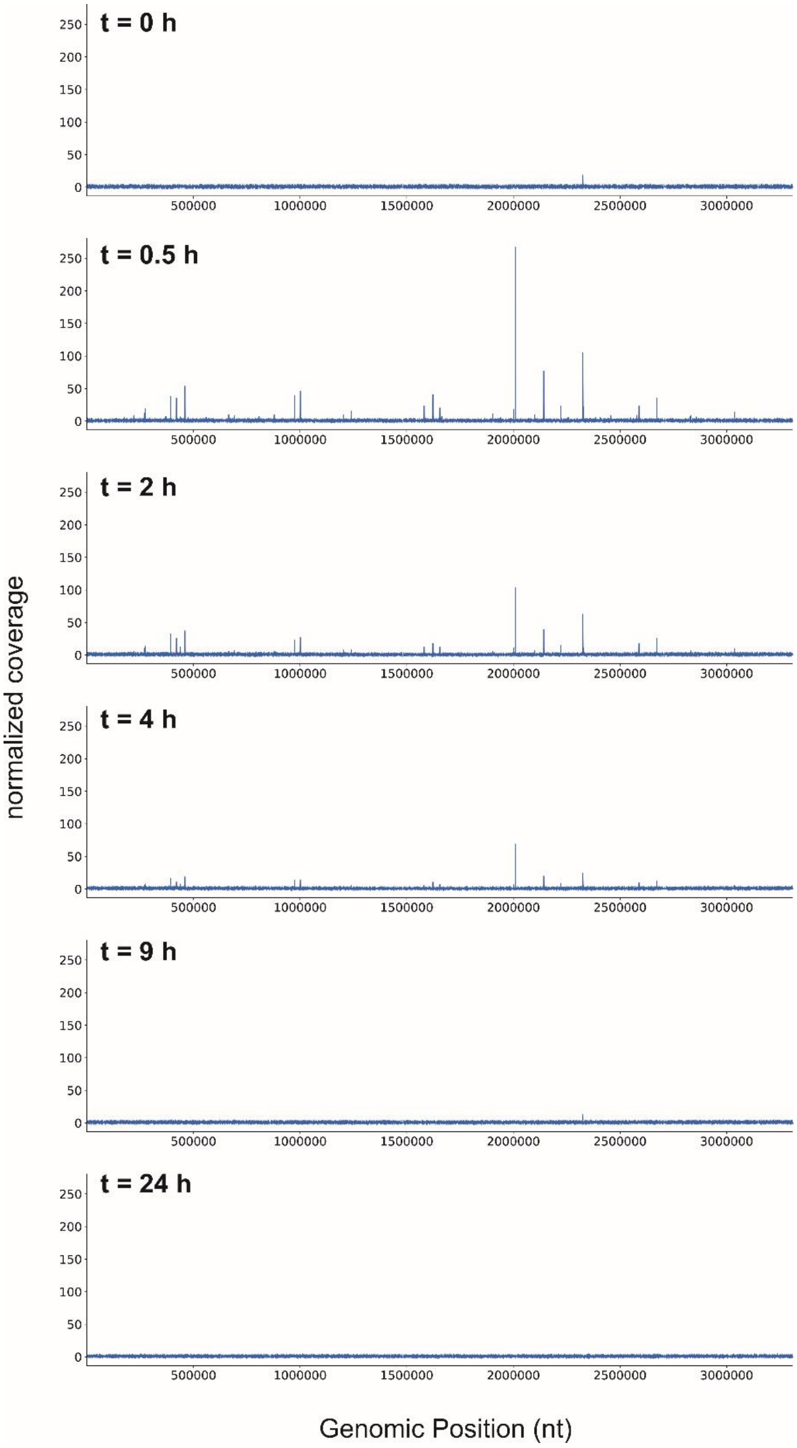

Figure S3: Global binding pattern of HrrA in the *C. glutamicum* genome in response to hemin addition. Genomic coverage (number of reads covering a particular genomic position) was normalized to the average coverage of the regions not harbouring binding peaks. Thus, depicted peak intensities are comparable between different time points. The strain *C. glutamicum*::*hrrA*-C-*twinstrep* was cultivated in CGXII minimal medium (lacking $FeSO_4$) supplemented with 2% (w/v) glucose and 4 µM hemin was added at 0 h. Cells were harvested at different time points as described in Figure 1.

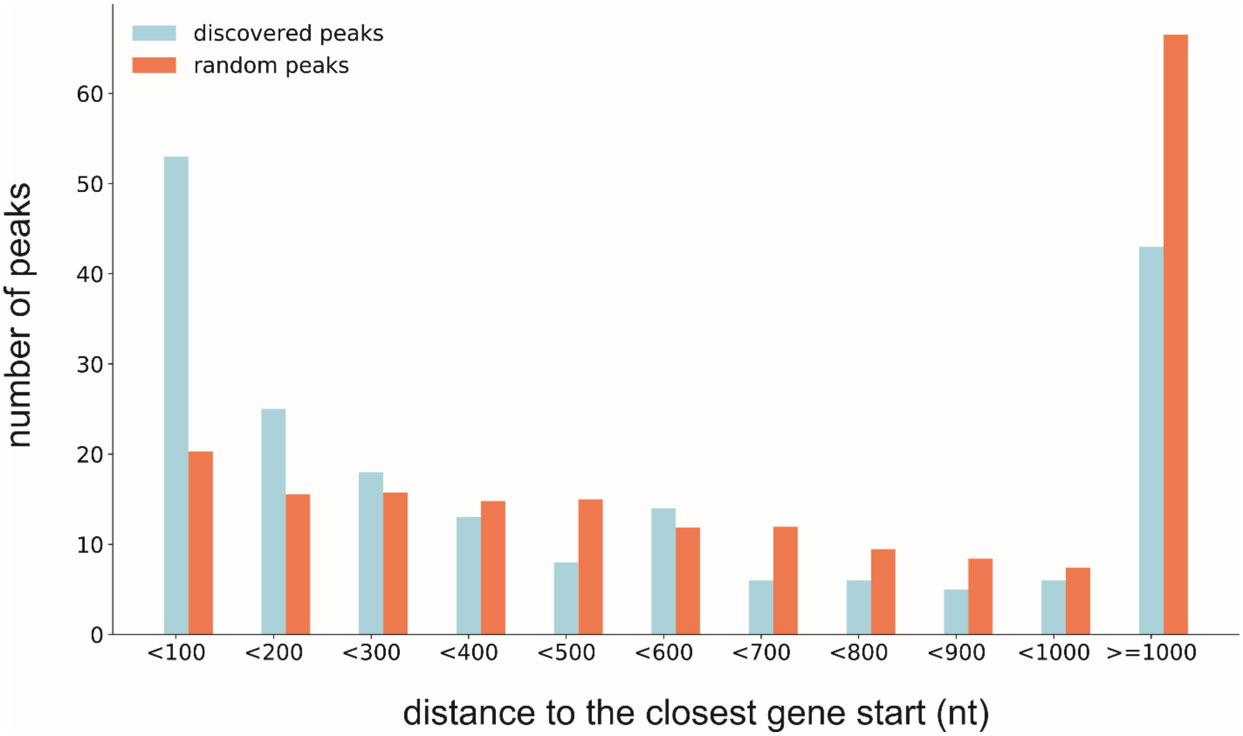

Figure S4: Distribution of distances from HrrA binding peaks centres to the closest downstream gene start site (transcription start site, TSS). As a background (red color), random peaks of the same width as real ones were generated. Random peak generation was performed 100 times and resulting distance distributions were then averaged into a single background distribution. Peaks with a distance of <100 nt can also be found up to 60 nt downstream of the TSS.

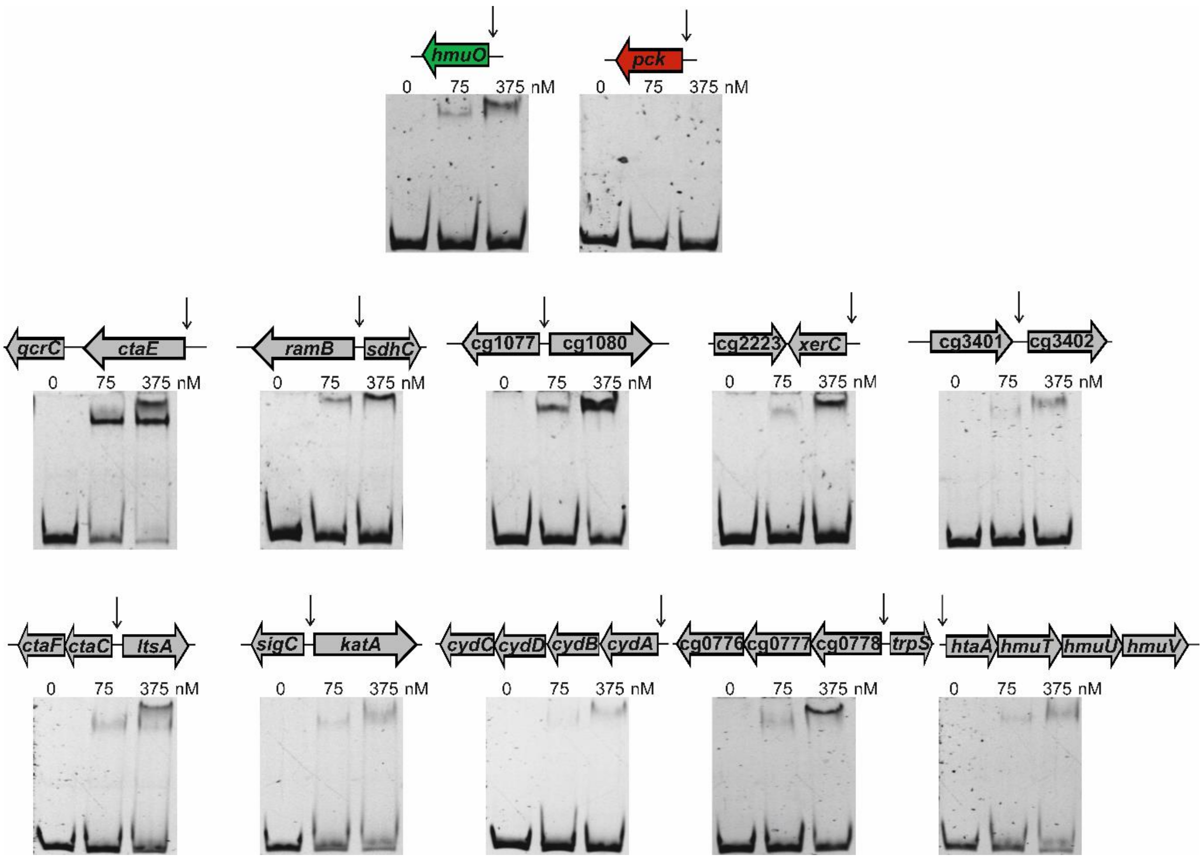

Figure S5: HrrA binding to selected target promotor regions. Protein-DNA interactions were validated by electrophoretic mobility shift assays (EMSA) using 15 nM DNA fragments covering 50 bp up- and downstream of the maximal ChAP-Seq peak height and an increasing protein monomer concentration of 0, 75 and 375 nM. The genomic location of the maximal peak height found in the ChAP-Seq experiments is indicated by an arrow. As control, the promoter regions of *hmuO* (positive control) and *pck* (negative control) were used.

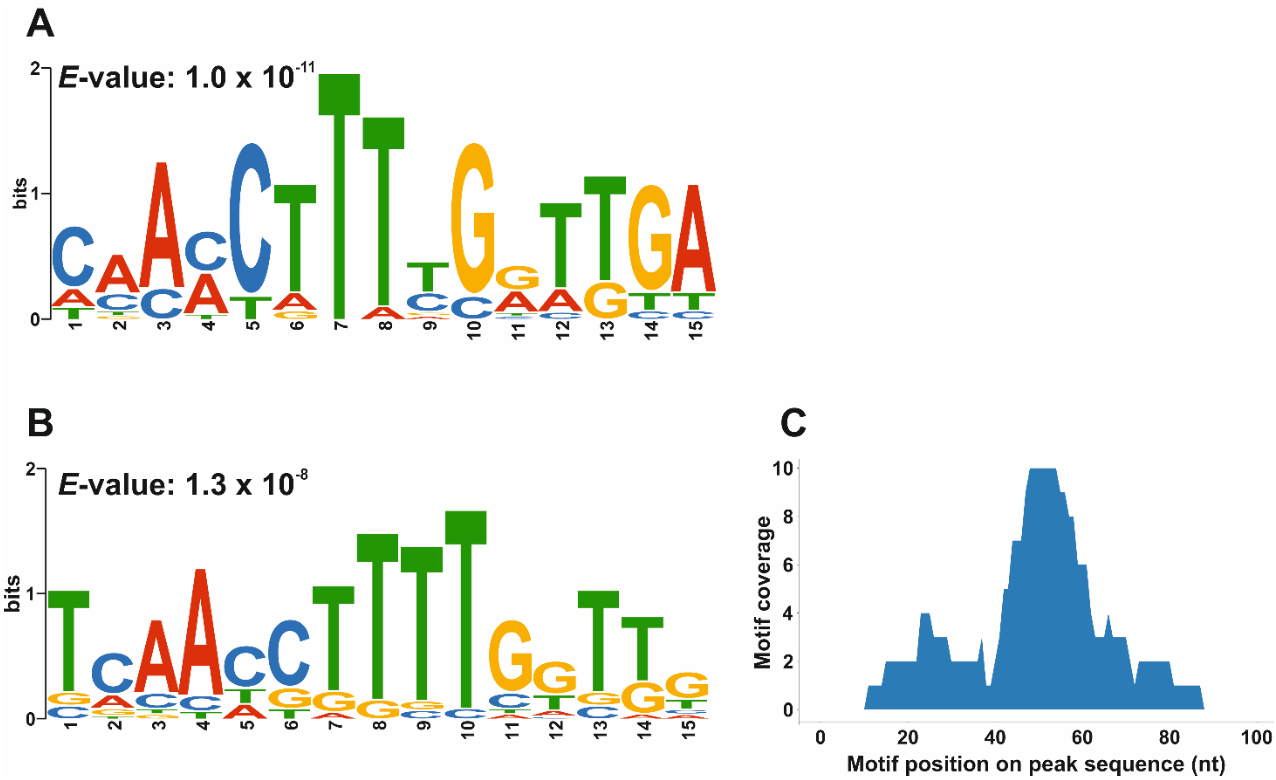

Figure S6: Derivation of a HrrA binding motif revealed a weakly conserved palindromic sequence. Sequences of all peaks with at least two-fold increased coverage (T_0) (A) or 100 bp of the tested EMSA DNA fragments (Figure S5) (B) were used for a MEME v.5 analysis (http://meme-suite.org). (C) Shown is the position of identified motif sequences within the analysed peak sequences used in (B). The majority of HrrA motifs centre at the position of the peak maximum (at 50 nt).

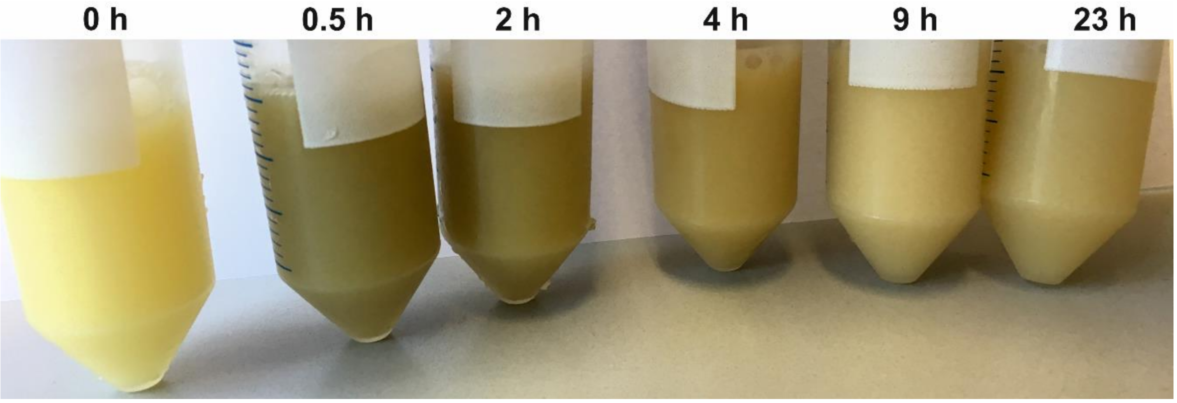

Figure S7: Visual inspection of *C. glutamicum* cells before and after addition of heme. Iron-starved *C. glutamicum* wild type cells were cultivated in CGXII medium (2 % (w/v) glucose, without $FeSO_4$) and cells were harvested at different time points before and after the addition of 4 µM heme. Cell pellets were subsequently resuspended in Tris buffer (100 mM Tris-HCl, 1 mM EDTA, pH 8.0) and adjusted to an OD_{600} of 3.5.

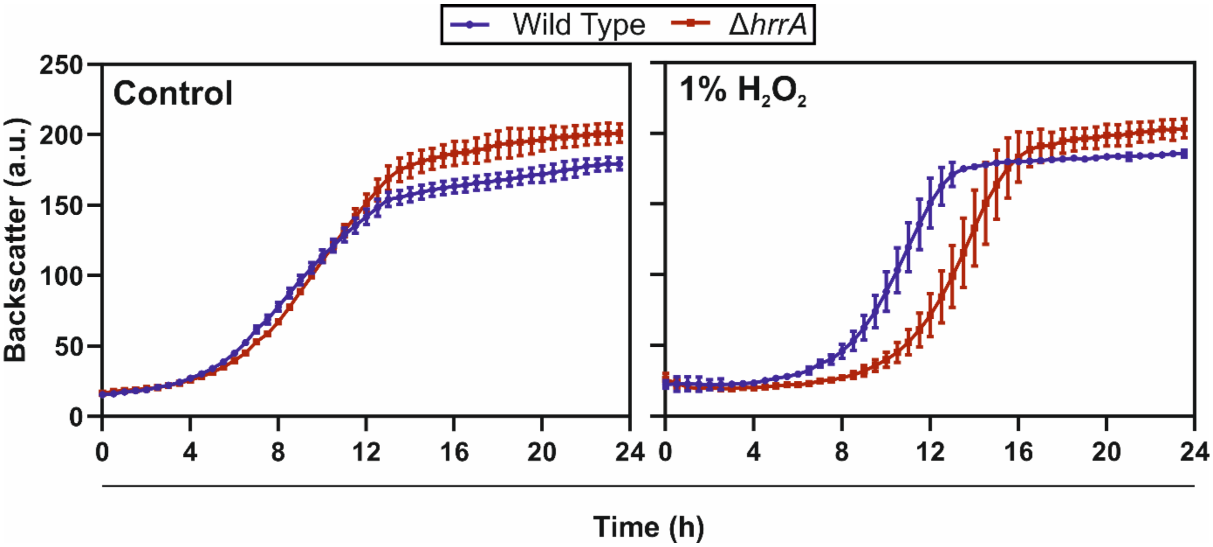

Figure S8: Growth assays revealed an increased sensitivity of *C. glutamicum* Δ*hrrA* against oxidative stress. Iron-starved *C. glutamicum* wild type as well as the mutant strain Δ*hrrA* were inoculated to an OD_{600} of 1 in CGXII medium (2% (w/v) glucose, 4 µM hemin, without $FeSO_4$) and subsequently incubated for 15 min at RT either with 1% (v/v) H_2O_2 or without. This incubation time of 15 min served as avoidance of misleading backscatter measurements due to foam generation. After the incubation, cells were transferred to microtiter plates and cultivated in a microbioreactor cultivation system. Growth curves shown are based on backscatter measurements (expressed in arbitrary units (a.u.)) of three biological replicates. The error bars represent the standard deviation of these replicates.

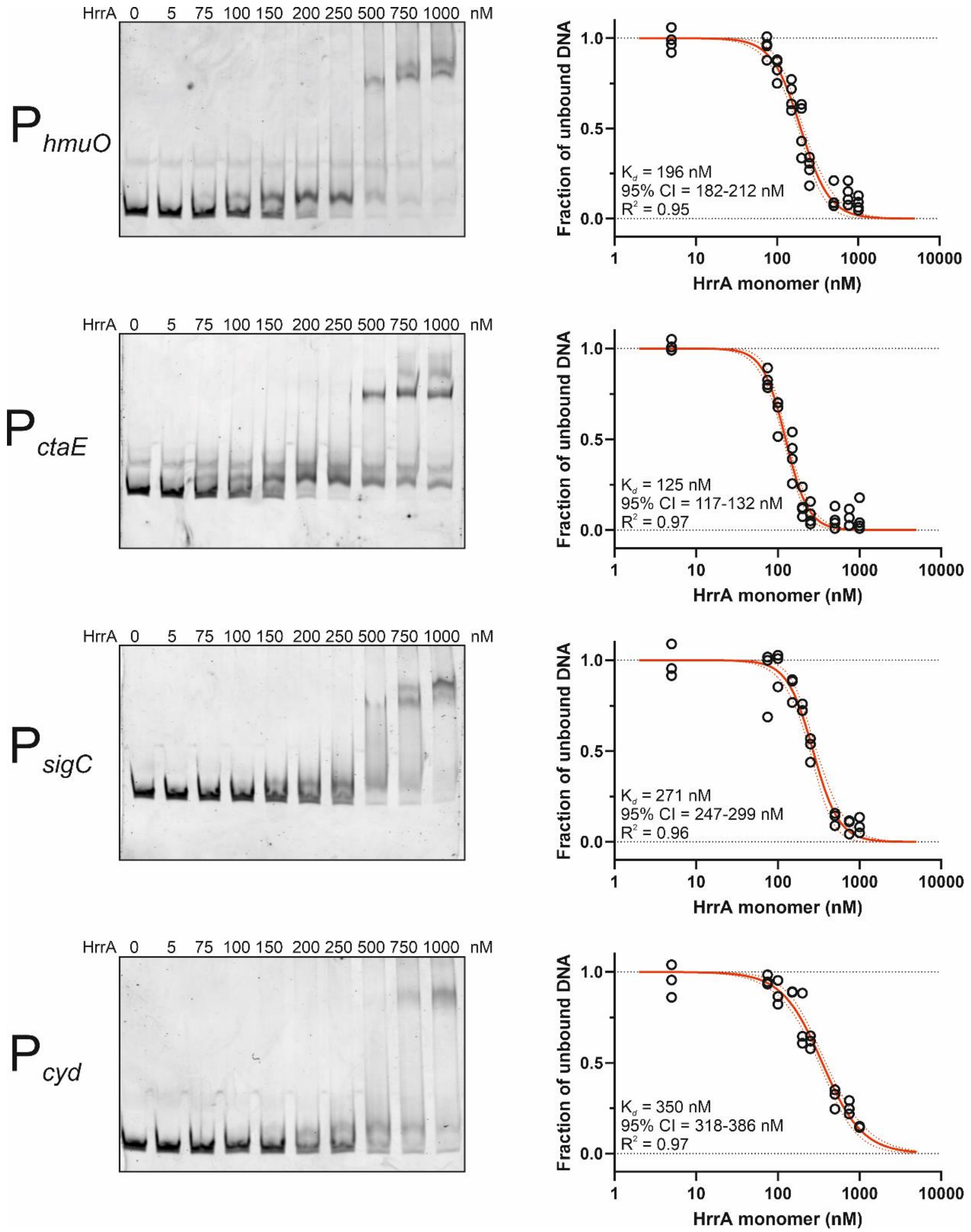

Figure S9: Binding affinity of HrrA to selected target promoters. Depicted are representative images of quantitative EMSAs used for analysis of protein-DNA interaction and the calculation of HrrA affinities to the different promoters. For the analysis, 10 nM Cy3-labelled 98-105 bp DNA fragments containing the maximal ChAP-Seq peak height were used with increasing amounts of HrrA (given as monomers). Determination of unbound DNA in EMSA studies allowed the calculation of HrrA binding affinities to different target promoters. Quantification of unbound DNA band intensities was performed using Image Studio Lite (Licor, Bad Homburg, Germany) and apparent K_d values were calculated using GraphPad Prism 7. The calculation of apparent K_d – values is based on 3-4 gels each. Black dotted lines represent top and bottom constraints for the fit. Red dotted lines represent the 95% confidence level. CI, confidence interval.

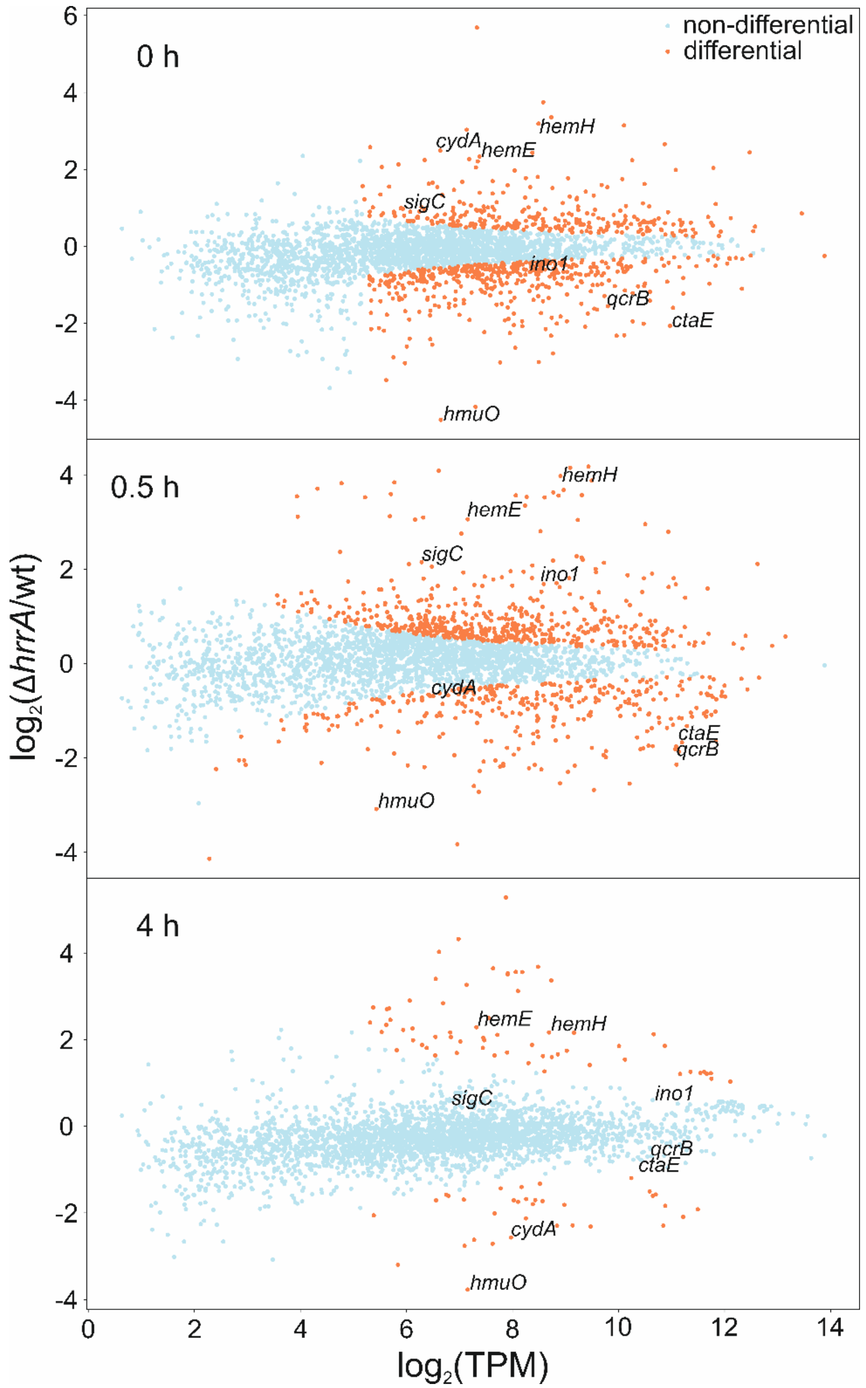

Figure S10: Time-resolved differential gene expression analysis. Shown is the $\log_2$ fold change in gene expression (Δ*hrrA* versus wild type) along with a $\log_2$ mean expression (expression averaged for Δ*hrrA* and WT samples) in transcripts per million (TPM). Orange dots represent significantly differentially expressed genes with an empirical FDR <0.05 (see material and methods). Wild type and Δ*hrrA C. glutamicum* strains were grown in CGXII medium (without $FeSO_4$) supplemented with 2% (w/v) glucose and 4 µM hemin (T_0 is prior addition of hemin; for details on cultivation and sample preparation see material and methods).

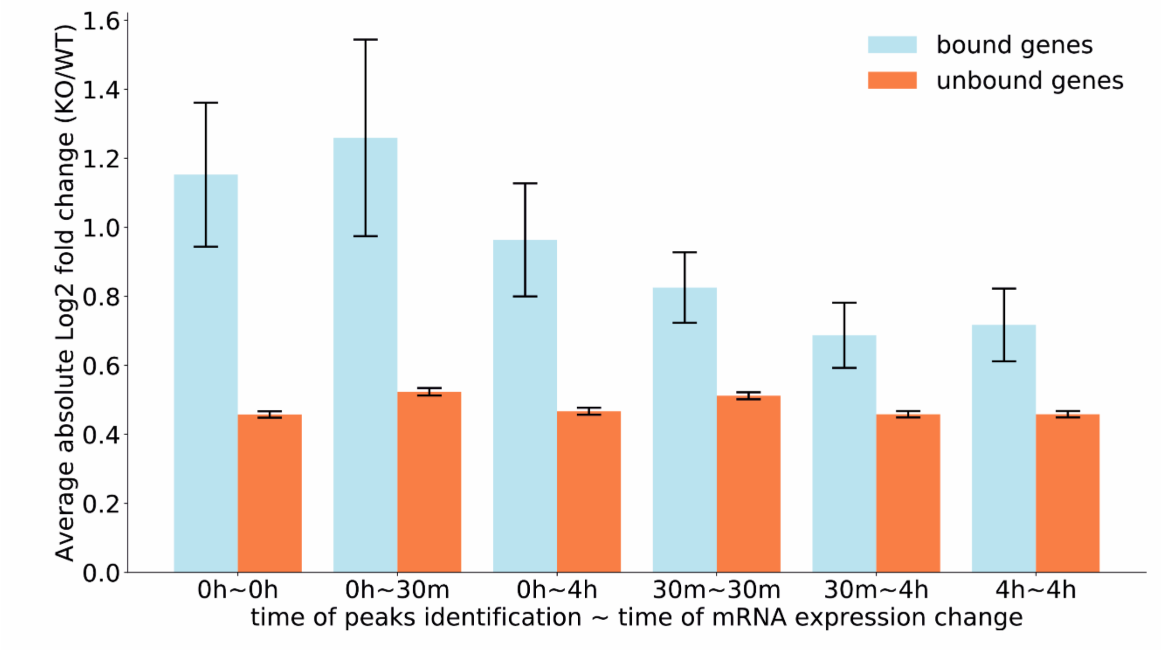

Figure S11: Correlation of HrrA binding and expression change. For the time-points 0h, 0.5h, 4h all the *C. glutamicum* protein-coding genes with decent expression (>10 TPM in Δ*hrrA* and WT samples) were split into groups: bound by HrrA (the ones which have an HrrA binding peak within 800 nt region upstream or 200 nt downstream to the transcription start site) and unbound. For these groups mean absolute log2 fold change (Δ*hrrA*/WT) was calculated for the time-points 0h, 0.5h, 4 h along with standard error of the mean.

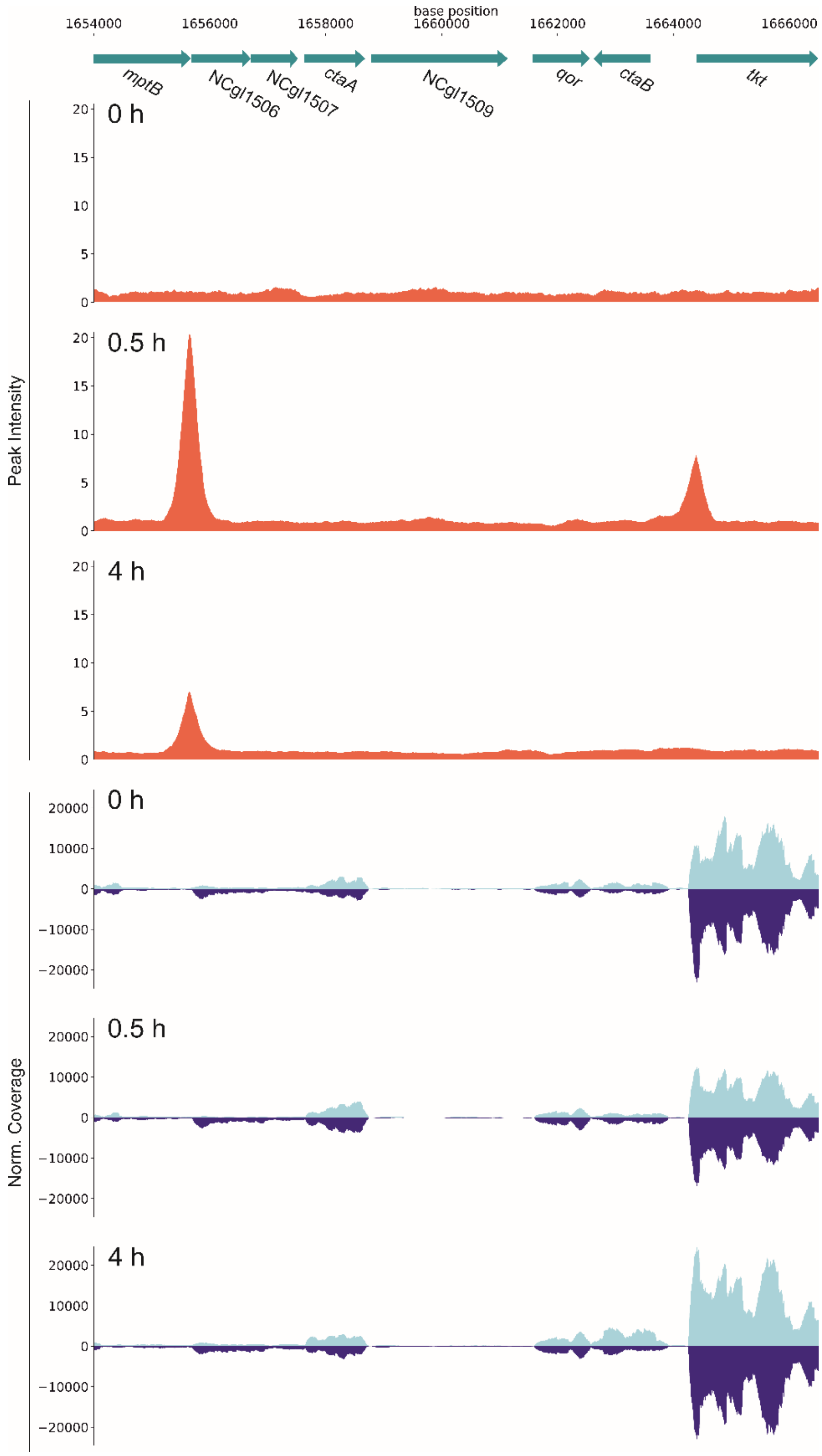

Figure S12: HrrA coordinates expression of *ctaA* and *ctaB* in response to heme. Shown are the ChAP-Seq (orange) and RNA-Seq (blue) results focusing on the *ctaA* and *ctaB* locus in the genome of *C. glutamicum.* Depicted is the genomic region between *mptb* (cg1766) and *tkt* (cg1774). For the cultivation, CGXII medium supplemented with 2% (w/v) glucose and 4 µM hemin was inoculated with iron starved cells from a stationary culture and adjusted to an OD_{600} of 3.5. Samples were analysed at the indicated time points as described in material and methods.

Supplementary Tables

Table S1: Bacterial strains and plasmids used in this study. Oligonucleotides used for the construction of the plasmids are listed in Table S2.

Strain	Relevant characteristics	Reference
Escherichia coli		
DH5α	*fhuA2 lac(del)U169 phoA glnV44 Φ80' lacZ(del)M15 gyrA96 recA1 relA1 endA1 thi-1 hsdR17*; for general cloning purposes	Invitrogen
BL21(DE3)	B F^{-} *ompT gal dcm lon* $hsdS_B(r_B^{-}m_B^{-})$ λ(DE3 [*lacI lacUV5-T7p07 ind1 sam7 nin5*]) [$malB^{+}$]$_{K-12}$($λ^S$); overexpression of proteins.	(1)
Corynebacterium glutamicum		
***C. glutamicum* ATCC 13032**	Biotin-auxotrophic wild type strain	(2)
C. glutamicum* Δ*hrrA	Derivative of ATCC 13032 with in-frame deletion of the *hrrA* gene (cg3247).	(3)
C. glutamicum*::*hrrA*-C-*twinstrep	Derivative of ATCC 13032 encoding a C-terminally twinstrep-tagged version of *hrrA* (cg3247).	This study

**Plasmids**		
**Name**	**Resistance**	**Source**
**pK19 *mob sacB***	Kanamycin	(4)
**pK19 *mob sacB_hrrA*-C-*twinstrep***	Kanamycin	This study

Table S2: Oligonucleotides used in this study.

#	Name	Sequence
**Construction of pK19 *mob sacB_hrrA*-C-*twinstrep***		
1	hrrA-LF-twin-strep_fw	CAAGCTTGCATGCCTGCAGGTCGACGCGGAATCGACGTCATCTTG
2	hrrA-LF-twin-strep_rv	ACCTAAAGCCTTGCAGCAACCCCCGCTATTTTTCGAACTGCGGGTGG
3	hrrA-RF_fw	GAGCCACCCGCAGTTCGAAAAATAGCGGGGGTTGCTGCAAGGC
4	hrrA-RF_rv	ATTCGAGCTCGGTACCCGGGGATCCCCGGAATCAATACACCGGC
**Amplification of DNA probes for EMSAs**		
5	P_{hmuO} (EMSA) fw	GAGAAATCCTCACGCTCAC
6	P_{hmuO} (EMSA) fw-Cy3	Cy3-GAGAAATCCTCACGCTCAC
7	P_{hmuO} (EMSA) rv	GGTGGGAGCCCCAAAGTTG
8	P_{ctaE} (EMSA) fw	CCCAAAGTGGTTTCCGCAGG
9	P_{ctaE} (EMSA) fw-Cy3	Cy3-CCCAAAGTGGTTTCCGCAGG
10	P_{ctaE} (EMSA) rv	ACGCCTTTTATTCGGGTTC
11	P_{pck} (EMSA) fw	CTTTCTATGGAGATGATCG
12	P_{pck} (EMSA) rv	CGATTTAAATGGACCCTAAAC
13	P_{ramB} (EMSA) fw	CCTGCGCAAAGTTGCTCCCTG
14	P_{ramB} (EMSA) rv	CTCACAGGATACCGATCCGAAC
15	P_{cg1080} (EMSA) fw	CGCTCCTCTGTGGGATTTGTC
16	P_{cg1080} (EMSA) rv	GCCTTCACTCCCTCAAAC
17	P_{xerC} (EMSA) fw	CTTAGGCTTGCCTCACACAC
18	P_{xerC} (EMSA) rv	AATGCGGAAATGCCATAAAACC
19	P_{cg3402}(EMSA) fw	CATAGGGGTATAGCCTTGAG
20	P_{cg3402}(EMSA) rv	CAGTGTGCGCAGGTCATGCC
21	P_{ctaC}(EMSA) fw	GGAATACCTAAAGTCTAGGC
22	P_{ctaC}(EMSA) rv	GTAGGAACGTAGGGGGTAAG
23	$P_{sigC/katA}$(EMSA) fw	GGTCACCATAAAGGTGTGTAG
24	$P_{sigC/katA}$(EMSA) fw-Cy3	Cy3-GGTCACCATAAAGGTGTGTAG
25	$P_{sigC/katA}$(EMSA) rv	GCCACCAAATAATCAGCCC
26	P_{cyd} (EMSA) fw	GTTCCCGCTCACAGCTTAAC
27	P_{cyd} (EMSA) fw-Cy3	Cy3-GTTCCCGCTCACAGCTTAAC
28	P_{cyd} (EMSA) rv	GGTGACTTGTCAACAAGGGG
29	P_{trpS} (EMSA) fw	GACTTGTTTACCCAAGCAATAC
30	P_{trpS} (EMSA) rv	CCGGTGAGGCAACATTTACC
31	P_{htaA} (EMSA) fw	GTCATGATGGCGTCTCGGGC
32	P_{htaA} (EMSA) rv	GTAATCAACGCACAAATG

Table S5: Pearson correlation for the gene expression values (TPM) between the two biological replicates. Transcriptome expression estimates for all the three time-points and both KO and WT conditions show high reproducibility. The genes with low expression (combined expression in replicates < 5 TPM) were not included in this analysis.

Time Point	WT	KO
**0 h**	0.9987	0.9994
**0.5 h**	0.9990	0.9984
**4 h**	0.9974	0.9964

References

1. Studier, F.W. and Moffatt, B.A. (1986) Use of bacteriophage T7 RNA polymerase to direct selective high-level expression of cloned genes. *J. Mol. Biol.*, **189**, 113-130.
2. Kalinowski, J., Bathe, B., Bartels, D., Bischoff, N., Bott, M., Burkovski, A., Dusch, N., Eggeling, L., Eikmanns, B.J., Gaigalat, L. *et al.* (2003) The complete *Corynebacterium glutamicum* ATCC 13032 genome sequence and its impact on the production of L-aspartate-derived amino acids and vitamins. *J Biotechnol*, **104**, 5-25.
3. Frunzke, J., Gätgens, C., Brocker, M. and Bott, M. (2011) Control of heme homeostasis in *Corynebacterium glutamicum* by the two-component system HrrSA. *J Bacteriol*, **193**, 1212-1221.
4. Schäfer, A., Tauch, A., Jager, W., Kalinowski, J., Thierbach, G. and Pühler, A. (1994) Small mobilizable multi-purpose cloning vectors derived from the *Escherichia coli plasmids* pK18 and pK19: selection of defined deletions in the chromosome of *Corynebacterium glutamicum*. *Gene*, **145**, 69-73.
